# Macrophages as orchestrators of gut-organ axis crosstalk: immunometabolic reprogramming and host-directed therapy in sepsis

**DOI:** 10.3389/fcimb.2026.1921762

**Published:** 2026-10-19

**Authors:** Wenqing Zeng, Ye Zhang, Liping Fan, Ying Xun, Zhaoqing Jiang, Feifei Dai

**Affiliations:** 1 Department of Intensive Care Medicine, the Second Affiliated Hospital of Zhejiang Chinese Medical University, Hangzhou, Zhejiang, China; 2 Department of Pharmacy, The Second Affiliated Hospital of Zhejiang Chinese Medical University, Hangzhou, Zhejiang, China; 3 Department of Clinical Laboratory, the Second Affiliated Hospital of Zhejiang Chinese Medical University, Hangzhou, Zhejiang, China; 4 Department of Endocrinology, Tongde Hospital of Zhejiang Province, Hangzhou, China

**Keywords:** gut-organ axis, host-directed therapy, immune-metabolism, macrophages, sepsis

## Abstract

Sepsis is a life-threatening syndrome driven by a dysregulated host response to infection. Among the numerous contributory factors, the gut microbiota and its metabolites are increasingly recognized as potential modulators of systemic inflammation, acting through multiple gut-organ axes. Within this network, macrophages serve as central integrators that transduce microbial signals into organ-specific inflammatory or reparative outcomes. Disease evolution is critically determined by immune-metabolic reprogramming of macrophages. This reprogramming comprises tricarboxylic acid cycle truncation, lipid remodeling, mitochondrial dysfunction, and inflammasome activation. These processes are closely intertwined with NFκB, cGAS-STING, TLR/MyD88, and aryl hydrocarbon receptor signaling. Gut-derived metabolites, particularly short-chain fatty acids and indole derivatives, shape macrophage functions in distal organs. These mechanisms predominantly operate during the acute phase of sepsis, driving early organ injury. In contrast, sepsis survivors often enter a distinct chronic phase, characterized by persistent immune dysregulation. Trained immunity, through metabolic-epigenetic rewiring, may underlie this long-term dysfunction in survivors. Preclinical promise has been demonstrated by host-directed therapies that target macrophage metabolism. Nevertheless, major challenges impede clinical translation. These include inadequate preclinical models, the absence of validated real-time biomarkers for immune-metabolic phenotyping, and marked heterogeneity among patients. This review positions macrophages as actionable sentinels in gut-organ crosstalk during acute sepsis, and further explores their role in chronic sequelae, underscoring the need for biomarker-guided stratification and precision immune-metabolic editing to overcome barriers in both settings.

## 1 Introduction

Sepsis, a life-threatening organ dysfunction due to an abnormal response to infection, presents a major global health challenge with high mortality rates (Rudd et al., 2020; Liu et al., 2022; GBD 2021 Antimicrobial Resistance Collaborators, (2024)). From 1990 to 2017, the age-standardized rates of sepsis incidence and mortality fell by 37% and 52.8%, respectively (Rudd et al., 2020). However, sepsis and septic shock are more frequent and deadly in China compared to North America and Europe (Liu et al., 2022). Viral pathogens like those causing dengue, COVID-19, and Ebola contribute to about 30% of sepsis cases (G Gürtler et al., 2025). A recent study presents a new analytical method to compare Centers for Disease Control and Prevention Adult Sepsis Event (CDC ASE), Sepsis-3, and International Classification of Diseases (ICD) standards for identifying sepsis in electronic health records, showing that patients meeting both CDC ASE and Sepsis-3 criteria were more severely ill with a 14% in-hospital mortality rate (Bauer et al., 2025).

Sepsis pathophysiology has evolved from earlier concepts of ischemia or bacterial toxicity to an immune disease caused by abnormal host responses (Wiersinga and van der Poll, 2022; Saavedra-Torres et al., 2025b). A biphasic immune cascade is initiated by the release of pathogen-associated molecular patterns (PAMPs) and damage-associated molecular patterns (DAMPs). It commences with a hyper-inflammatory phase that serves to clear pathogens. However, this phase frequently overlaps with progressive immune suppression, resulting in a chaotic response (Saavedra-Torres et al., 2025b). In this context, DAMPs are not merely bystanders of tissue injury. They are now recognized as independent triggers of macrophage metabolic reprogramming, capable of activating inflammatory pathways even in the absence of microbial stimuli (Kim et al., 2025).

Sepsis can lead to a chronic state of immune dysregulation, characterized by a persistent immune remnant (PIR). This is outlined in the Sepsis-Associated Persistent Multiorgan Immunometabolic Syndrome (SIMMP-Sepsis model), where survival marks the beginning of a prolonged phase of active, but maladaptive, immune restoration rather than the end of the disease (Saavedra-Torres et al., 2025b). These maladaptive changes increase susceptibility to secondary infections and drive end organ damage, culminating in multi organ dysfunction (Wiersinga and van der Poll, 2022; Saavedra-Torres et al., 2025a).

The immune-metabolic derangements described are mainly confined to the acute phase of sepsis, when enteric signals and macrophage reprogramming drive early organ injury (O'Neill et al., 2016; Malla et al., 2025; Torres et al., 2025; Du et al., 2026; Liu et al., 2026; Zhang et al., 2026). Survivors, however, often transition to a chronic phase featuring persistent immune dysregulation, combined metabolic and epigenetic rewiring, and heightened susceptibility to secondary infections (Gorecki et al., 2025; Lahariya et al., 2025; Torres et al., 2025). These two pathological states, acute organ dysfunction and postseptic immune sequelae, differ fundamentally in their mechanistic basis, temporal profile, and therapeutic relevance, and therefore should be distinguished (Liu and Liang, 2025; Torres et al., 2025; Zhu and He, 2026).

The M1/M2 polarization paradigm posits a metabolic dichotomy. Pro-inflammatory responses in classically activated M1 macrophages are supported by aerobic glycolysis. Conversely, tissue repair in alternatively activated M2 macrophages is driven by fatty acid oxidation (FAO) and oxidative phosphorylation (OXPHOS). However, this binary classification is a useful heuristic method that fails to capture the heterogeneity of macrophages *in vivo*, as cells exist in a dynamic continuum influenced by local signals and individual genetics. Thus, the framework demands context-sensitive, dynamic refinement (Galvan-Pena and O'Neill, 2014; Van den Bossche et al., 2015; Zhao et al., 2025). Acting as sentinel cells in organs such as the gut, liver, and lungs, these macrophages detect microbial products and adapt their functional states in response to signals from the microbiota and injured tissues (Gao et al., 2023). Their metabolic plasticity is central to inter-organ communication via the gut-organ axes. Hence, therapeutic strategies to re-establish immune equilibrium are warranted. In sepsis, these metabolic pathways become disrupted, concurrently driving both inflammation and immunosuppression.

The gut microbiome is a critical driver of immune metabolic dysregulation, but it is not the exclusive source of metabolic signals. Sepsis rapidly induces gut dysbiosis and impairs intestinal barrier integrity. Consequently, translocation of PAMPs is facilitated, and the profile of microbiota-derived metabolites is altered. Altered metabolites include short-chain fatty acids (SCFAs) and tryptophan derivatives (Ney et al., 2023; Tian and Li, 2025; Meng et al., 2026; Zhao et al., 2026; Zheng et al., 2026). Gut-derived signals are sensed by tissue-resident macrophages in distant organs. This sensing subsequently triggers a cascade of immune-metabolic reprogramming. The balance between organ protection and injury is ultimately dictated by this reprogramming (Fang et al., 2026; Liu et al., 2026). However, the crosstalk is bidirectional. Septic tissue injury induces the release of DAMPs. Macrophage metabolic reprogramming is potentiated by these molecules. Consequently, a self-amplifying loop is established. This loop perpetuates the inflammatory response and exacerbates organ damage (Kim et al., 2025; Yang et al., 2025; Zhang et al., 2026). Thus, metabolic reprogramming in macrophages arises from at least two converging inputs: microbial products (PAMPs and microbiota derived metabolites) and host derived danger signals (DAMPs) (Kim et al., 2025; Zhang et al., 2026). The gut microbiota is now recognized as a central contributor-though not the sole driver-of sepsis pathophysiology, modulating systemic inflammation and orchestrating multi-organ dysfunction through the gut-liver, gut-lung, gut-brain, gut-heart, and gut-kidney axes (Tang et al., 2025; Wang et al., 2025).

In parallel, emerging evidence indicates that sepsis survivors exhibit a distinct immune-metabolic phenotype (Gorecki et al., 2025; Saavedra-Torres et al., 2025a). Trained immunity (TRIM) is characterized by the metabolic and epigenetic rewiring of myeloid cells. This reprogramming may represent a mechanistic driver of the persistent hyper-inflammatory or immunosuppressive states observed in this patient cohort (Kim et al., 2025; Xu et al., 2026). This long-term reprogramming is not a mere extension of the acute insult but rather a separate adaptive response that predisposes to recurrent infections and late mortality (Wang et al., 2023; Sviridov et al., 2025). The role of macrophages in this chronic phase remains less defined, but preliminary data suggest that gut-microbiota alterations persist and may contribute to sustained immune dysregulation (Kim et al., 2025).

Substantial progress has been made in immune-metabolism research. Nevertheless, clinical applications remain limited for both immune-metabolic reprogramming and host-directed therapy for sepsis. This limitation is particularly evident when macrophages are targeted as central regulators of the gut-organ axis. This is due to simplistic models and a lack of personalized treatments. The traditional biphasic model of sepsis posits an initial hyper-inflammatory phase followed by immunosuppression. However, this model is overly simplistic, because both phases can occur concurrently (Wiersinga and van der Poll, 2022). Current preclinical models fail to adequately capture human diversity. Factors such as comorbidities and antimicrobial exposure are often overlooked. Moreover, no host-directed sepsis therapy has been approved to date (Strich et al., 2026). This failure is partially attributed to insufficient stratification of patient immune status (Rao et al., 2025). Our knowledge of gut-organ interactions and metabolic changes across organs and disease stages remains incomplete. In addition, the translation of multi-omics into clinical biomarkers is still in its infancy. These challenges collectively emphasize the urgent need for precise immune-metabolic profiling and patient stratification to enable effective sepsis therapies.

This review critically examines macrophages as pivotal regulators of gut-organ communication in sepsis. Special emphasis is placed on immune-metabolic reprogramming. A coherent mechanistic framework is proposed, grounded in evidence from experimental and clinical studies. The framework starts with infection-induced gut dysbiosis and barrier disruption. Gut-derived signals, including microbial products and metabolites, are then transmitted to distant organs. These signals subsequently trigger metabolic and functional reprogramming of resident macrophages. This reprogramming, in turn, determines the balance between organ-specific injury and protection during the acute phase. In parallel, DAMPs released from injured tissues contribute as an independent, non-microbial signal that further shapes macrophage metabolism and amplifies the inflammatory response. Beyond the acute response, long-term sequelae such as TRIM in survivors increasingly valued, although evidence remains limited. A synthesis of pertinent experimental and clinical findings reported in recent years is provided in **Table 1**.

**Table 1:** Research on host-directed therapy for sepsis based on immune metabolism reprogramming.

Author/year/[ref.]	Types of animal/participants	Types of therapeutic targets	Intervention	Data of laboratory or clinical	Damaged organs	Effect (conclusion)
Yang Z et al./2026/ (Yang et al., 2026)	Male C57BL/6 mice	NFκB/NLRP3/Caspase-1 signaling pathway	Dachaihu decoction	Laboratory	Gut-liver axis	Positive
Yu T et al/2026/ (Yu et al., 2026)	Male C57BL/6 mice; patients with sespsis and healthy volunteers	Gut microbiota- UDCA- Macrophage axis; TLR4/NFκB signaling pathway	Simiao Yongan decoction	Laboratory and clinical	Gut-liver axis	Positive
Wang et al./2023/ (Wang et al., 2023)	Male C57BL/6J mice; Patients	PXR	IPA	Laboratory and clinical	Gut-liver axis	Positive
Murao A et al./2025/ (Murao et al., 2025)	C57BL/6 J mice	Neutrophil elastase and PAR-1	IELs	Laboratory	Gut-liver axis	Positive
Wang L et al./2025/ (Wang et al., 2026)	Male C57BL/6 mice	AMPK	GDF15	Laboratory	Liver	Positive
Wei M et al./2026/ (Wei et al., 2026)	C57BL/6J mice and healthy individuals	Gut microbiota	KD	Laboratory and clinical	Gut-lung axis	Positive
Zhao L et al./2026/ (Zhao et al., 2026)	C57BL/6 mice	Gut microbiota and intestinal barrier	gut microbiota-derived SCFAs	Laboratory	Gut-brain axis	Positive
Huang ZB ZG et al./2025/ (Huang et al., 2025)	C57BL/6 WT mice and sepsis patients in ICU	Gut microbiota	IAA	Laboratory and clinical	Gut-brain axis	Positive
Tang J et al./2026/ (Tang et al., 2026a)	Sprague Dawley female rats	The RORγt/IL-17 pathway	ALY688	Laboratory	Gut- heart axis	Positive
Sun X. et al./2022/ (Sun et al., 2022)	C57BL/6 mice	Tnfsf10	miR-24-3p from M2-exo	Laboratory	Heart	Positive
Gao H et al./2023/ (Gao et al., 2023)	Vsig4^-/-^ mice (C57BL/6J-Vsig4^em1Smoc^)	Gut microbiota derived mEVs	Vsig4+ macrophages	Laboratory	Gut- heart axis	Positive
Zhang J et al./2024/ (Zhang et al., 2024b)	Wild-type (WT) C57BL/6 mice	Mincle/syk/NFκB signaling	Fer-1; SAP130	Laboratory	Kidney	Positive
Tian X et al./2025/ (Tian et al., 2025)	C57BL/6 mice	STING-GSDMD axis	SCFA	Laboratory	Gut- kidney Kidney	Positive
Li F et al.*/*2025/ (Li et al., 2025)	Male C57BL/6J mice	Glycolytic inhibitor	2-DG	Laboratory	Lung	Positive
Uehara I et al./2022/ (Uehara et al., 2022)	C57BL/6, SKG (CREA Japan, Tokyo, Japan), and Il-6-/– mice	Glycolytic inhibitor	2-DG	Laboratory	Lung	Positive
Wang Y et al.*/*2026/ (Wang et al., 2026)	Male C57BL/6 wild-type mice	Glycolytic inhibitor	CARM1 inhibitor TP-064; Shikonin	Laboratory	Lung	Positive
Wang J et al./2026/ (Wang et al., 2026)	C57BL/6J mice	PKM2-NLRP3 inhibitor	PD3	Laboratory	Lung	Positive
Huang J et al./2026/ (Huang et al., 2026)	*Lyz2-cre-Scap^f/f^* mice; C57BL/6J mice	PKM2, a key glycolysis enzyme	Scap, DASA58	Laboratory	Unspecified	Positive
Ji T et al./2025/ (Ji et al., 2025)	Male C57BL/6J mice	Mitochondrion; mtROS/cGAS-STING-IFN-β pathway	Metformin	Laboratory	Unspecified	Positive
Sun J et al./2025/ (Sun et al., 2025)	C57BL/6 mice	Mitochondrion	MitoQ	Laboratory	Lung	Positive
Mohsin M et al./2025/ (Mohsin et al., 2025)	C57BL/6 mice	Mitochondrion	Urolithin-A	Laboratory	Lung	Positive
Manfredini A CL et al.*/*2019/ (Manfredini et al., 2019)	Wistar rat	Mitochondrion	Rosiglitazone, rapamycin and rilmenidine	Laboratory	Brain	Positive
Li X et al./2024/ (Li et al., 2024)	Sprague-Dawley rats	Autophagy	Rapamycin, 3-Methyladenine	Laboratory	Lung, liver, and kidney	Positive
He S et al./2024/ (He et al., 2024)	Male C57BL/6 mice	Promoting M2 macrophage polarization via the SIRT1/NFκB pathway	NMN	Laboratory	Lung	Positive
Hong G et al./2018/ (Hong et al., 2018)	C57BL/6 mice	NAD+/SIRT1 signaling	NR	Laboratory	Lung and heart	Positive
Zhao GJ et al./2023/ (Zhao et al., 2023)	Male C57BL/6 mice	NAD+/SIRT1 pathway	NR	Laboratory	Lung, heart, liver, and kidney	Positive
Cros C et al.*/*2022/ (Cros et al., 2022)	C57BL6 mice	Reprogramming of macrophages from M1 to M2	β-NMN	Laboratory	heart and lung	Positive
Li HR et al./2023/ (Li et al., 2023)	C57BL/6J mice	SIRT1	NMN	Laboratory	Brain	Positive
Arioz Tunc H et al./2026/ (Arioz Tunc et al., 2026)	healthy infants	Enhance SCFAs	Prebiotics including GOS, FOS, 2FL, and yeast β-glucan	Clinical	Gut-lung axis	inconsistent
Zhao Z et al./2025/ (Zhao and Wu, 2025)	Male C57BL/6J mice	Gut microbiota- NET- integrin axis	FMT	Laboratory	Gut-lung axis	Positive
Tang Y CL et al./2024/ (Tang et al., 2024)	C57BL/6 mice	Improve intestinal flora and increase beneficial metabolites, fatty acids and secondary bile acids	FMT	Laboratory	Gut-lung axis	Positive
Wang MT et al./2026/ (Wang et al., 2026)	C57BL/6J mice	Gut microbiota homeostasis and mitochondrial function	FMT	Laboratory	Gut-lung axis	Positive
Yang Y et al./2024/ (Yang et al., 2024)	a 69-year-old male	Gut microbiota homeostasis	WMT	Clinical	Unspecified	Positive
Qin H et al./2024/ (Qin et al., 2024)	severe scrub typhus with pneumonia, sepsis, and multiple organ dysfunction in a pregnant woman	Gut microbiota homeostasis	FMT and oral fecal microbiota capsule therapy	Clinical	Unspecified	Positive
Wang LT et al./2023/ (Wang et al., 2023)	C57BL/6 mice	Tissue-resident and bone marrow (BM)-recruited macrophage (MΦ) polarization	PMSCs	Laboratory	Lung	Positive
Matthay MA ZH et al./2025/ (Matthay et al., 2025)	120 ventilated patients with ARDS (PaO2/FiO2 ratio <250 mm Hg)	/	MSCs	Clinical	Lung	Negative
Ye R et al./2025/ (Ye et al., 2025)	115 sepsis patients; Male Sprague–Dawley rats	The MEF2C/Hippo signaling pathway	miR-223-3p in LPS-EVs	Laboratory and clinical	Lung	Positive
Chen F et al./2026/ (Chen et al., 2026)	Specific pathogen free male C57BL/6 mice	NFκB/NLRP3 signaling pathway	MSC-sEV	Clinical	Kidney	Positive

AMP-activated protein kinase, AMPK; 2-deoxy-d-glucose, 2-DG; 2’-fucosyllactose, 2FL; β- nicotinamide mononucleotide, β-NMN; extracellular vesicles, EVs; Fecal microbiota transplantation, FMT; Ferrostatin-1, Fer-1; Fructooligosaccharides, FOS; Gut intraepithelial lymphocytes, IELs; Growth differentiation factor 15, GDF15; galactooligosaccharides, GOS; Hypoxia-inducible factor 1α, HIF-1α; Human placental mesenchymal stromal (stem) cells, PMSCs; Intensive Care Unit, ICU; 3-indoleacetic acid, IAA; indole-3-propionic acid, IPA; Ketogenic diet, KD; Lipopolysaccharide, LPS; M2 macrophages, M2; Myocyte Enhancer Factor 2 C, MEF2C; Mesenchymal stromal (stem) cells, MSCs; Nicotinamide mononucleotide, NMN; Nicotinamide riboside, NR; Protease-activated receptor-1, PAR-1;Plantainoside D, PD; Activate Pregnane X receptor, PXR; Small extracellular vesicles, sEV; Spliceosome-associated protein 130, SAP130; Short-chain fatty acids, SCFAs; Ursodeoxycholic acid, UDCA; Toll-like receptor 4, TLR4; Washed microbiota transplantation, WMT.

## 2 Macrophages as mediators of gut-organ axis communication

The gut-organ axis concept posits that the intestinal ecosystem affects remote organs, with macrophages acting as key transducers. This section systematically examines five major axes based on the latest research evidence, with a particular attention to the gut driving mechanisms and systemic sepsis induced macrophage responses (**Figure 1**).

**Figure 1:**
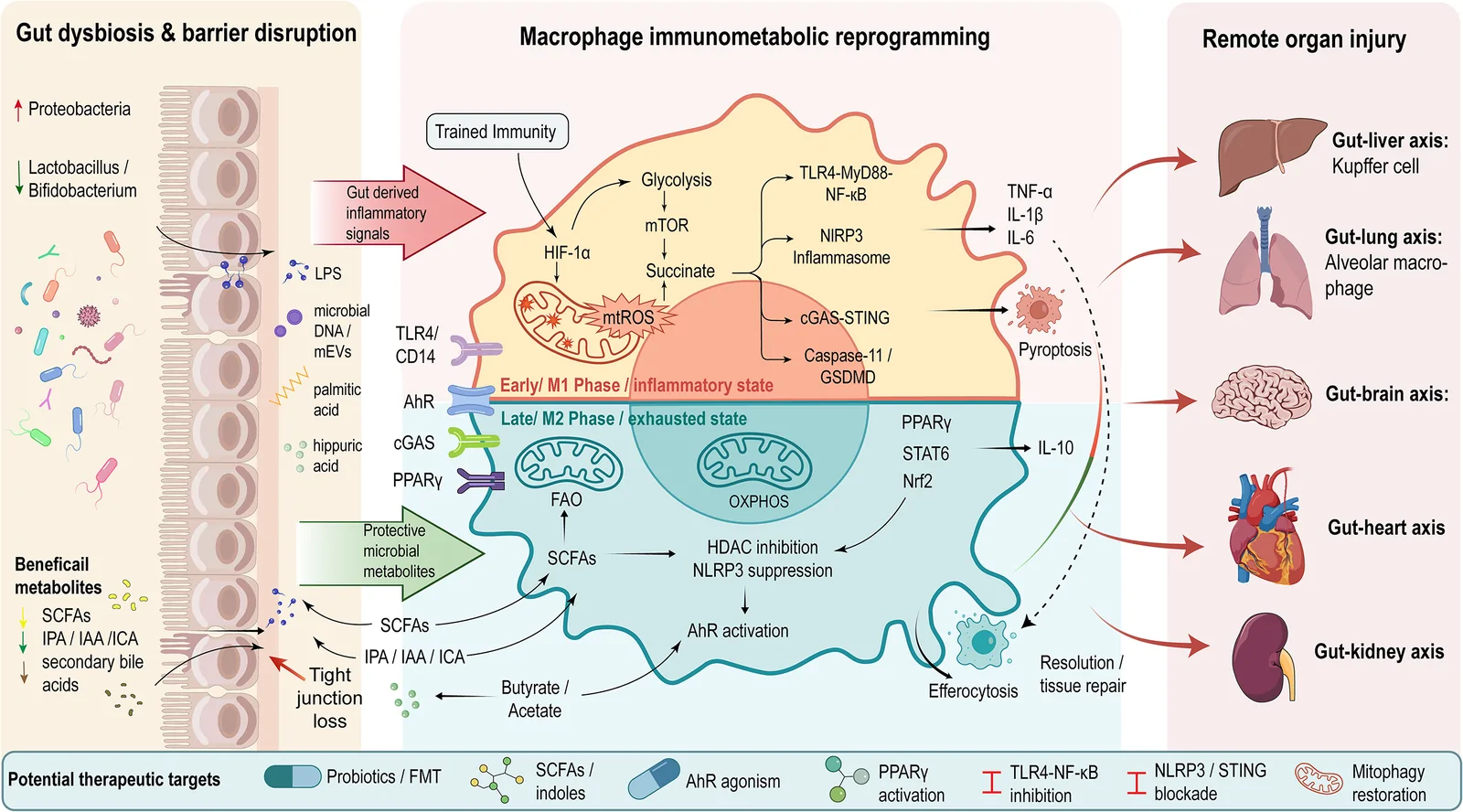
Schematic of gut dysbiosis-driven macrophage immunometabolic reprogramming and multi-organ dysfunction. Gut dysbiosis and barrier disruption facilitate the translocation of gut-derived inflammatory signals (LPS, microbial DNA, mEVs) while depleting protective metabolites (SCFAs, IPA/IAA/ICA, secondary bile acids), thereby initiating macrophage immunometabolic reprogramming. In the early M1/pro-inflammatory phase, trained immunity and enhanced glycolysis, mTOR, and succinate signaling activate TLR4-MyD88-NFκB, the NLRP3 inflammasome, and cGAS-STING, culminating in TNF-α/IL-1β/IL-6 secretion and Caspase-11/GSDMD-mediated pyroptosis. Conversely, the late M2/exhausted phase features FAO/OXPHOS via AhR/PPARγ activation and histone deacetylase (HDAC) inhibition, which suppresses NLRP3, promotes IL-10 production and efferocytosis, and facilitates resolution via STAT6 and Nrf2. This immunometabolic imbalance drives multi-organ dysfunction across the gut-liver, gut-lung, gut-brain, gut-heart, and gut-kidney axes. The bottom panel indicates potential therapeutic strategies, including probiotics/FMT, AhR agonism, PPARγ activation, TLR4-NFκB inhibition, NLRP3/STING blockade, and mitophagy restoration.

### 2.1 The gut-liver axis

Hepatic macrophages positioned at the portal-central venous gradient serve as primary sentinels for gut-derived signals. Direct experimental evidence from murine models demonstrates that lipopolysaccharide (LPS) activates macrophages through Toll-like receptor 4 (TLR4), triggering inflammatory cascades that contribute to hepatic injury (Zhang et al., 2022; Jain et al., 2024). Conversely, clinical observations in patients with porto- sinusoidal vascular disorder reveal elevated serum LPS correlated with increased TLR4-positive macrophages, providing indirect associations supporting the gut-liver-macrophage axis in human pathology (Gioia et al., 2023; Overi et al., 2025).

Gut dysbiosis and barrier disruption permit bacterial products, especially LPS, to reach the liver and activate Kupffer cells and recruited macrophages via signaling eliciting a cytokine storm that damages hepatocytes and correlates with adverse outcomes (Sun et al., 2020; Chen et al., 2024; Yang et al., 2026; Yu et al., 2026). It directly points out the concept of the Gut microbiota-ursodeoxycholic acid (UDCA)-macrophage axis, which provides a promising integrated treatment strategy for sepsis induced liver injury (SLI) (Yu et al., 2026). Additionally, dysbiosis-induced activation of intestinal epithelial Myd88 during sepsis suppresses FXR-FGF15 signaling, which exacerbates bile acid derangement and impairs intestinal barrier function (Qian et al., 2025). Protective microbial metabolites, such as indole-3-propionic acid and SCFAs, decline during sepsis, thereby removing anti-inflammatory restraints on macrophages (Ney et al., 2023; Wang et al., 2023). Macrophages further drive metabolic dysfunction through impaired FAO and mitochondrial stress, while gut-primed neutrophils release extracellular traps that augment Kupffer cell activation (Murao et al., 2025).

However, the conventional M1/M2 framework inadequately captures macrophage heterogeneity. Intravital imaging and zonal transcriptomics reveal that periportal Marco^+^ macrophages, induced by isoallolithocholic acid from Odoribacteraceae, suppress inflammation via IL-10 and scavenger receptor sequestration; their disruption in Marco deficiency or chronic liver diseases including primary sclerosing cholangitis (PSC) and non-alcoholic steatohepatitis (NASH) exacerbates hepatic inflammation (Miyamoto et al., 2024). Growth differentiation factor 15 alleviates sepsis-associated liver injury by promoting protective macrophage polarization via AMP-activated protein kinase (AMPK)-mediated inhibition of Pyruvate kinase M2 (PKM2)/hypoxia-inducible factor-1α (HIF-1α) -driven glycolysis (Wang et al., 2026). Additionally, SCFAs such as butyrate limit inflammatory macrophage niches in non-alcoholic steatohepatitis by inducing a pro-healing phenotype (Sarkar et al., 2023).

These findings underscore that macrophage function is shaped by dynamic interactions among microbial signals, metabolic states, and anatomical positioning. Therapeutic strategies targeting macrophage immune-metabolism, including approaches that restore gut microbial homeostasis and reprogramme macrophages towards anti-inflammatory states, offer promising avenues for treating liver diseases arising from gut-liver axis dysregulation.

### 2.2 The gut-lung axis

In sepsis, the gut-lung axis constitutes a bidirectional ecological and immune network connecting the intestinal and pulmonary microbiota (Yuan et al., 2026). Its pathological features include intestinal barrier failure, translocation of microbial products, and immune cell trafficking, all of which promote lung injury. Alveolar macrophages integrate these signals via pattern recognition and metabolic reprogramming, thus linking microbial ecology to pathogen clearance and inflammatory resolution to sustain host defense and immune homeostasis (Yuan et al., 2026).

The metabolic reprogramming and functional polarization of alveolar macrophages are critically shaped by gut derived signals such as endotoxin translocation and SCFAs depletion (Fan et al., 2025; Li et al., 2025; Maruyama et al., 2026). The causal sequence is supported by multiple lines of direct experimental evidence. Sepsis-associated acute respiratory distress syndrome (ARDS) is characterized by macrophage metabolic reprogramming, with a glycolytic M1 phenotype driven by HIF-1α that fuels pro-inflammatory cytokine release and tissue injury, versus an oxidative phosphorylation/FAO-dependent M2 phenotype that promotes resolution and repair (Li et al., 2024; Liu et al., 2025; Tang et al., 2025; Tang et al., 2026b). Moreover, HIF-1α is regulated by oxygen-dependent hydroxylation and inflammatory cascades (NFκB, PI3K/Akt/mTOR, STAT3), and in pulmonary epithelial cells it protects via Foxm1-mediated repair, Fas/FasL anti-apoptosis, and glycolysis to offset mitochondrial dysfunction (Liu et al., 2025). Sepsis- induced Wnt/β- catenin signaling upregulates C-C motif chemokine ligand 1 (CCL1), recruiting gut-derived γδ T17 cells that produce IL-17A and worsen acute lung injury (ALI) (Xie et al., 2024). Engineered probiotics delivering IL-1Ra reinforce the intestinal barrier and modulate pulmonary macrophage polarization, providing direct proof of gut-lung communication (Yao et al., 2025).

Recent studies have shown that gut metabolites add further regulation. Dietary fiber promotes propionate, which producing gut microbiota that metabolically reprogram alveolar macrophages to attenuate sterile lung inflammation yet aggravate infectious injury. This effect is transferable via fecal microbiome transplantation (FMT), underscoring the gut-lung axis in governing resting pulmonary immunometabolic tone (Maruyama et al., 2026). A ketogenic diet enriches L. reuteri and L. plantarum to yield azelaic acid, which expands MerTK^+^ alveolar macrophages via Peroxisome proliferator-activated receptor gamma (PPAR-γ), enhancing efferocytosis and resolution (Wei et al., 2026). Manganese-doped carbon dots boost indole-3-propionic acid, activating the aryl hydrocarbon receptor on macrophages to promote anti-inflammatory polarization, thereby attenuated septic lung injury (Peng et al., 2025). Conversely, microbiota-derived palmitic acid activates Nicotinamide Adenine Dinucleotide Phosphate oxidase 2 (NOX2)/NOD-like receptor family pyrin domain-containing 3 (NLRP3), driving macrophage pyroptosis and mitigating lung injury-a pathway blocked by platinum-doped emodin carbon dots (Pt-ECDs) (Song et al., 2025).

Macrophages act as central hubs that integrate microbial, metabolic, and cellular signals to shape lung outcomes in sepsis. Interventions targeting this axis show promise, but the context-dependent and dynamic nature of macrophage responses demands patient stratification and biomarker development for personalized therapy.

### 2.3 The gut-brain axis

The gut-brain axis critically contributes to sepsis-associated encephalopathy (SAE), with microglia-the brain’s resident macrophages-as central mediators (Zhang et al., 2025). Gut barrier disruption allows systemic translocation of LPS and other microbial products, which, together with circulating cytokines, compromise the blood-brain barrier and activate microglia (Zhang et al., 2024a; Payal et al., 2025). Sustained M1-like polarization and NLRP3 inflammasome activation in microglia directly instigate neuroinflammation and consequent neuronal injury (Xiao et al., 2025; Zhang et al., 2025). Gut microbial metabolites shape these responses: SCFAs mitigate microglial NLRP3 activation via NLR family pyrin domain containing 6 (NLRP6) signalling, attenuating hippocampal inflammation and cognitive deficits (Zhao et al., 2026). Conversely, the tryptophan metabolite 3-indoleacetic acid (IAA), reduced in SAE patients, activates microglial aryl hydrocarbon receptors (AhRs) to suppress pro-inflammatory cytokines; IAA supplementation improves SAE phenotypes in murine models (Huang et al., 2025). Despite these insights, the precise programming of macrophage-microglial crosstalk by gut-derived signals remains incompletely defined, and selective therapies targeting this axis are scarce. Thus, while microglia are recognized as key effectors in gut -brain signaling in sepsis, mechanistic knowledge gaps persist, necessitating additional translational investigations (Wei et al., 2025).

### 2.4 The gut-heart axis

Macrophages are increasingly recognized as key effectors in the gut-heart axis during sepsis induced cardiomyopathy (Hobai, 2023; Zhou et al., 2025; Wu et al., 2026). ALY688 activated macrophages to promote Treg differentiation and strengthened intestinal barrier to restrict LPS leakage, supporting gut-heart axis modulation via macrophages in sepsis-associated cardiac injury (Tang et al., 2026a). Sepsis induced dysbiosis alters short chain fatty acids and trimethylamine oxide, contributing to myocardial dysfunction (Xiao et al., 2023). Additionally, M2 macrophage exosomal miR-24-3p alleviates myocardial injury by inhibiting Tnfsf10 (Sun et al., 2022). Moreover, reduced Vsig4^+^ macrophages permit gut microbial DNA to reach cardiomyocytes via vesicles and activate cyclic GMP-AMP synthase (cGAS)-stimulator of interferon genes (STING), driving cardiac dysfunction that is reversible by macrophage restoration (Gao et al., 2023). Collectively, these studies establish macrophages as central integrators of gut-derived signals that influence cardiac outcome in sepsis, and highlight modulation of this gut-heart axis as a promising therapeutic avenue for sepsis-induced cardiomyopathy.

### 2.5 The gut-kidney axis

Sepsis-induced gut dysbiosis promotes LPS translocation, which activates renal macrophages via the TLR4/NFκB pathway and drives tubular injury (Huang et al., 2025). Renal dysfunction subsequently impairs gut barrier integrity, establishing a bidirectional vicious cycle that positions macrophages as central effectors and supports microbiome-targeted therapeutic strategies for sepsis-associated acute kidney injury (SA-AKI). Within this self-perpetuating loop, ferroptotic tubular epithelial cells release SAP130, which polarises macrophages toward the M1 phenotype via Mincle/syk/NFκB signalling; these M1 macrophages in turn exacerbate tubular ferroptosis (Zhang et al., 2024b). Conversely, gut-derived butyrate was depleted in SA-AKI, and its supplementation attenuated renal injury by suppressing STING- Gasdermin D (GSDMD)-dependent NLRP3 pyroptosis in mice and HK-2 cells; STING overexpression reversed this effect, providing direct evidence for the gut-kidney-macrophage axis (Tian et al., 2025). Additional regulatory molecules have recently been identified: Kruppel-like factor 6 (KLF6) upregulation represses ornithine decarboxylase 1 (ODC1) transcription and promotes macrophage pyroptosis (Ding et al., 2025), whereas myeloid cells-1 (TREM1) deficiency favours protective M2 polarisation through activation of the PI3K/AKT and signal transducer and activator of transcription 6 (STAT6) pathways (Xu et al., 2026). Collectively, macrophage immunometabolism and gut-derived signals critically determine SA-AKI progression.

## 3 Immunometabolic reprogramming of macrophages in sepsis

Macrophage immunometabolic reprogramming is central to sepsis pathogenesis, which involving glycolytic shift, tricarboxylic acid (TCA) cycle truncation, lipid remodeling, and crosstalk between mitochondria and inflammasomes.

### 3.1 M1/M2 polarization

In sepsis, classically activated M1 macrophages undergo glycolytic reprogramming controlled by epigenetic, transcriptional, and metabolic mechanisms. Epigenetically, SET Domain-Containing 2 (SETD2) mediates H3 lysine 36 trimethylation (H3K36me3) to regulate *Hif1a*, which in turn inhibits M1 macrophage polarization and glycolysis (Meng et al., 2023). At the transcriptional level, activating transcription factor 4 (ATF4) is elevated in septic patients’ peripheral blood mononuclear cells (PBMCs), correlating with glucose metabolism and activating a pro-inflammatory pathway in macrophages through mTOR (Liu et al., 2023). ATF4 overexpression increases pro-inflammatory cytokines, lactate, and hexokinase 2 (*HK2*) in LPS-tolerant macrophages, highlighting ATF4 as a key glycolytic activator that enhances inflammation and decreases in macrophages during sepsis, suggesting its potential as a target for sepsis immunotherapy (Liu et al., 2023). In septic cardiomyopathy, LPS-upregulated *Dancr* enhances *HK2* expression through *insulin-like growth factor 2 mRNA binding protein 2 (IGF2BP2)*-mediated mRNA stabilization, increasing M1 glycolysis; *Dancr* silencing reduces M1 activity and improves cardiomyocyte viability, identifying this axis as a therapeutic target (Ma et al., 2025). The glycolytic inhibitor 2-deoxy-D-glucose (2-DG) enhances survival in cecal ligation and puncture (CLP) -induced septic mice by promoting neutrophil migration to infection sites. It achieves this by reducing G protein-coupled receptor kinase 2 (GRK2) expression and restoring C-X-C motif chemokine receptor 2 (CXCR2) levels, with the CXCR2 antagonist SB225002 partially hindering this effect (Tan et al., 2020). Collectively, these findings demonstrate that HIF-1α-driven glycolytic reprogramming in M1 macrophages is regulated through various mechanisms, and targeting this pathway could be a promising therapy for sepsis.

The polarization of macrophages toward the anti-inflammatory M2 phenotype is intrinsically linked to a distinct metabolic program characterized by elevated OXPHOS, enhanced FAO, and robust mitochondrial biogenesis (Yang et al., 2025). Unlike pro-inflammatory M1 macrophages that rely on aerobic glycolysis (the Warburg effect), M2 macrophages utilize FAO as a major carbon source for the TCA cycle, thereby supporting sustained anti-inflammatory and tissue-repair functions (Chen et al., 2024; Yang et al., 2025). This metabolic adaptation is governed by a network of transcription factors and coactivators, including PPAR-γ), which coordinate the expression of genes involved in mitochondrial function and lipid metabolism (Tu et al., 2023; Yang et al., 2025). The reliance of M2 macrophages on intact mitochondrial metabolism is further underscored by the finding that glutamine supports M2 polarization through the suppression of sirtuin 5 (SIRT5)-mediated desuccinylation of pyruvate dehydrogenase, thereby replenishing OXPHOS (Zhu et al., 2022). Pharmacological or genetic interventions that promote FAO or inhibit glycolysis have been shown to skew macrophages toward the M2 phenotype. For instance, esculetin treatment in septic models enhances fatty acid β-oxidation in macrophages while suppressing glycolysis, leading to rebalanced M1/M2 polarization and alleviation of ALI (Chen et al., 2024). Similarly, irisin drives M2 differentiation by activating JAK2-STAT6-dependent transcriptional upregulation of PPARγ and nuclear factor erythroid-2-related factor 2 (Nrf2), thereby reinforcing the anti-inflammatory metabolic state (Tu et al., 2023). In the context of sepsis-induced organ injury, targeting immunometabolic checkpoints has demonstrated therapeutic promise. Activation of PPARγ by pioglitazone ameliorates sepsis-related liver injury via promoting M2 polarization through the PPARγ/IκBα/NFκB pathway (Yang et al., 2025). Moreover, Trifluoromethoxyphenyl propionylpiperidin urea (TPPU, an inhibitor of sEH) reprograms hepatic macrophages toward an M2-like phenotype by modulating arachidonic acid metabolism (Li et al., 2025). Collectively, these findings establish that immune-metabolic reprogramming, specifically the shift from glycolysis to FAO/OXPHOS, is not merely a consequence but a driver of M2 macrophage polarization in sepsis. Harnessing this metabolic plasticity offers a promising avenue for developing precision therapies aimed at restoring immune homeostasis and mitigating organ dysfunction in patients with sepsis.

### 3.2 The TCA cycle and immunometabolites

In sepsis, the TCA cycle of macrophages undergoes profound metabolic reprogramming that critically determines inflammatory outcomes. A well-recognized feature in pro-inflammatory macrophages is the truncation of the TCA cycle at two distinct enzymatic breaks: isocitrate dehydrogenase (IDH) and succinate dehydrogenase (SDH) (Wang et al., 2021). The study points out that this dual breakpoint leads to the accumulation of citrate, which is exported for itaconate synthesis, and succinate, which stabilizes HIF-1α and promotes an inflammatory phenotype (Tannahill et al., 2013). Mechanistically, nitric oxide (NO) generated by inducible nitric oxide synthase (iNOS) serves as a pivotal upstream regulator that modulates succinate and itaconate, thereby orchestrating metabolic reprogramming toward a pro-inflammatory state in macrophages (Bailey et al., 2019). Acute activation induces itaconate synthesis, thereby inhibiting SDH to mediate immune tolerance, while β-glucan-induced TRIM counteracts this immunosuppressive effect by suppressing immune-responsive gene 1 (IRG1) and upregulating SDH expression (Domínguez-Andrés et al., 2019). Beyond itaconate, other TCA intermediates exert context-dependent functions during sepsis. α-Ketoglutarate (α-KG) exhibits dual roles: it can limit inflammation by upregulating the scavenger receptor scavenger receptor (SR-A5) via alkB homolog 5 (ALKBH5)- mediated m^6^A demethylation (Liang et al., 2024), yet under certain conditions it is recognized as a critical immunometabolic checkpoint that triggers inflammatory cell death via the Ten-eleven translocation 2 (TET2)- absent in melanoma 2 (AIM2) signaling cascade (Li et al., 2026). Glutamine sustains the IRG1/itaconate axis to restrain NLRP3 inflammasome activation and pyroptosis in macrophages, and glutamine deprivation exacerbates inflammatory injury in septic mice (Chen et al., 2025). Moreover, glucocorticoid treatment in LPS- induced sepsis restores TCA cycle integrity while suppressing JAK1/STAT3 signaling, thereby reducing organ injury and mortality (He et al., 2025). Collectively, these findings establish that TCA cycle remodeling- particularly the IDH- itaconate- SDH- succinate axis is a central determinant of macrophage polarization and immune dysfunction in sepsis, offering multiple nodes for therapeutic intervention.

### 3.3 Lipid metabolism and cholesterol remodeling

During sepsis, macrophage immune-metabolic reprogramming is driven by lipid metabolism and cholesterol remodeling. Latest research suggests that Lipid droplets (LDs) play a crucial role in innate immunity by aiding bacterial clearance and coordinating a protective proinflammatory response during sepsis, relying on diacylglycerol acyltransferases (DGAT)-1 and Type I IFN mechanisms (Pereira-Dutra et al., 2026). Conversely, sepsis-induced mortality factor 4-like protein 1 (MORF4L1, also called MRG15) upregulation in hepatocytes promotes proprotein convertase subtilisin/kexin type 9 (PCSK9) secretion, skewing macrophages toward an M1-like phenotype and worsening liver injury (Gu et al., 2024). Mitochondrial β-oxidation, controlled by carnitine palmitoyl transferase 1 (CPT1), removes oxylipins and modulates leukocyte functions (Misheva et al., 2022). Adipose-derived mesenchymal stromal cells redirect macrophages toward an M2-like phenotype via AKT/mTOR-PPARγ signaling, promoting LD biogenesis and PGE2 synthesis while suppressing pro-inflammatory cytokines (Souza-Moreira et al., 2019).

Cholesterol homeostasis disruption and tissue remodeling are associated with sepsis prognosis (Hofmaenner et al., 2022). The microbial metabolite hippuric acid promotes cholesterol biosynthesis and M1-like polarization via TLR-MyD88 signaling, and its circulating levels correlate with sepsis mortality (Mirji et al., 2026). Ferroptosis is another metabolic pathway: in septic lungs, extracellular cold-inducible RNA-binding protein (eCIRP) reduces glutathione peroxidase 4 (GPX4) activity and increases lipid reactive oxygen species (ROS), linking metabolic stress to cell death (Shimizu et al., 2022). LPS tolerance involves defective deposition of active histone marks at promoters of lipid metabolism and phagocytosis genes; this effect is reversed by β-glucan through permissive epigenetic modifications at distal enhancers (Novakovic et al., 2016). Macrophages lacking TLR4 upregulate Abca1, enhancing cholesterol efflux and suppressing glycolysis, which promotes M2 polarization and reduces inflammation while altering endothelial crosstalk (Fu et al., 2025). Endothelial injury is mediated by signals from macrophages and by endothelial TLR4 activity (Fu et al., 2025). In human cholesterol-ester transfer protein (CETP) transgenic mice, CETP drives M2 polarization, inflammation, and dyslipidemia (elevated VLDL cholesterol, reduced HDL cholesterol), exacerbates emphysema caused by elastase, and supports a pathogenic role in chronic obstructive pulmonary disease (COPD) (Santana et al., 2021).

Thus, lipid metabolism and cholesterol remodeling are central to macrophage immunometabolic reprogramming in sepsis and represent actionable therapeutic targets.

### 3.4 Mitochondrial function and the inflammasome

Sepsis is characterized by a failure to restore immune homeostasis, a process in which macrophages undergo profound immunometabolic reprogramming that inextricably links mitochondrial function to inflammasome activity (Liao et al., 2026; Liu et al., 2026). An early shift from OXPHOS to glycolysis is necessary for rapid effector responses, but its sustained engagement, alongside impaired FAO, perpetuates inflammatory signaling and mitochondrial dysfunction, unleashing ROS that drive multi-organ injury (Liu et al., 2026). This dysfunctional state is mechanistically connected to mitochondrial metabolite; for instance, itaconate, an IRG1-derived mitochondrial metabolite, reprograms immunometabolism by inhibiting SDH and activating Nrf2, thereby modulating inflammatory and oxidative stress responses via NFκB, NLRP3, and JAK/STAT signaling (Gao et al., 2025). In addition, enhancing Sirt1-mediated deacetylation of p62 restores mitophagy, reduces mitochondrial ROS, and suppresses pro-inflammatory responses, highlighting how removing damaged mitochondria curbs inflammasome activation (Wang et al., 2025). Non-canonical inflammasome pathways are also directly targeted by metabolites; taurodeoxycholic acid (TDCA) confers protection against endotoxemia by suppressing the caspase-11/GSDMD pyroptotic pathway in macrophages (Ruan et al., 2026). The persistent metabolic and inflammatory stress can further induce long-lasting epigenetic changes, with DAMPs riving a TRIM state through the mTOR/HIF-1α axis and enhancer priming, which may underpin maladaptive responses in chronic critical illness (Kim et al., 2025). In contrast, negative regulators can subvert this protective metabolic response; elevated Protein Regulator of Cytokinesis 1 (PRC1) impairs host defense by suppressing both NFκB/JAK-STAT signaling and the infection-induced glycolytic reprogramming necessary for bactericidal function (Wei et al., 2026). Re-establishing metabolic control is therapeutically viable, as non-invasive 650 nm red-light therapy preserves mitochondrial membrane potential and reduces ROS via the adiponectin-AdipoR1 axis, which in turn curtails inflammatory macrophage recruitment and promotes a reparative phenotype (Zhang et al., 2026a). Collectively, these findings position the mitochondrial-inflammasome circuit as a central determinant of macrophage fate in sepsis, where metabolic intermediates, quality control pathways, and redox signals converge to either propagate or resolve pathological inflammation.

### 3.5 Trained immunity

TRIM drives long-lasting metabolic and epigenetic reprogramming of innate immune cells (Kim et al., 2025). The study suggests that damage-associated molecular patterns (e.g., HMGB1, mitochondrial DNA) induce TRIM via the mTOR/HIF-1α axis, leading to the Warburg effect, succinate accumulation, and enhancer priming marked by H3K4me1/H3K27ac in sepsis (Kim et al., 2025). This maladaptive innate memory contributes to chronic inflammation and secondary immunosuppression in sepsis survivors. In addition, this TRIM links acute sepsis to chronic cardiometabolic disorders such as atherosclerosis, insulin resistance and dyslipidemia (Lahariya et al., 2025). TRIM, together with TLR signaling, isolevuglandin adducts, and dyslipidemia, sustains persistent pro-inflammatory and immunosenescent states, which drive pro-fibrotic remodeling in cardiovascular and renal beds and elevate long-term morbidity after critical care (Owen et al., 2022).

Recent studies have established the gut microbiome-TRIM circuit as a key driver of chronic inflammation (Yang et al., 2025). In inflammatory bowel disease, gut-bone marrow axis signalling reprograms hematopoietic progenitors, sustaining intestinal inflammation (Pires and Longman, 2026). Microbial metabolites like butyrate exert context-dependent dual effects, modulating epigenetic and metabolic training of myeloid cells (Zhao et al., 2026). In sepsis, increased microbiome exposure via gut-derived extracellular vesicles improves host outcomes (Zhao et al., 2026). These findings highlight the therapeutic potential of targeting this circuit.

## 4 Key signaling pathways and receptors

### 4.1 NFκB/TGF-β signaling pathways

NFκB/TGF-β signaling mediate gut-organ axis crosstalk in sepsis. Gut microbiota and their metabolites orchestrate sepsis outcomes by steering macrophage polarization through NFκB and TGF-β signaling, forming a functional gut-organ axis. In septic intestinal injury, Lactobacillus murinus-derived indoleacetaldehyde inhibits NFκB-driven M1 and promotes TGF-β-driven M2 polarization, preserving barrier integrity (Yan et al., 2026). Bacterial indole-3-propionic acid (IPA) suppresses macrophage IL-1β via methionine metabolism and NFκB inhibition (Han et al., 2025), whereas Desulfovibrio vulgaris (enriched in sepsis) exacerbates multi-organ injury by enhancing NFκB/MAPK phosphorylation (Wu et al., 2025). Along the gut-kidney axis, high humidity enriches Meyerozyma caribbica that produces syringic acid, blocking NFκB and macrophage pyroptosis (Cai et al., 2026). Along the gut-liver axis, UDCA inhibits TLR4/NFκB-mediated macrophage polarization (Yu et al., 2026), and along the gut-lung axis, Xuanfei Baidu Decoction restores microbiota, reduces M1 polarization of macrophages, and downregulates NFκB (Yang et al., 2025b). Thus, NFκB and TGF-β in macrophages serve as central hubs translating gut microbial signals into organ protection or injury, offering promising therapeutic targets in sepsis.

### 4.2 The cGAS- STING pathway

cGAS-STING signaling reprograms macrophage metabolism by enhancing glycolysis and suppressing oxidative phosphorylation, thereby sustaining inflammatory responses. In this context, macrophages act as central sensors of gut-derived signals, linking microbial translocation to inflammation in distant organs through the cGAS-STING axis. Results indicate that the STING signaling pathway can contribute to lethal sepsis by promoting intestinal epithelial cells (IECs) apoptosis and through disrupting the intestinal barrier (Hu et al., 2019). Along the gut-brain axis, migrating small intestinal γδ T17 cells activate microglial cGAS-STING-C1q, driving synaptic pruning in SAE (Wu et al., 2025). In addition, melatonin (Mel) inhibition of cGAS-STING attenuated renal injury and enhanced autophagosome formation in SA-AKI mice (Cao et al., 2025). Cytosolic mtDNA activates the cGAS-STING axis and subsequently triggers NLRP3 inflammasome activation in S-AKI, suggesting that reducing mtDNA accumulation or inhibiting this axis may serve as a therapeutic target (Luo et al., 2024). Thus, macrophage cGAS-STING serves as a key checkpoint in gut-organ crosstalk, offering a unifying therapeutic target for sepsis and related disorders.

### 4.3 TLR/MyD88 signaling pathway

Gut-derived signals propagate along the gut-organ axis largely through the TLR-MyD88 signaling hub in macrophages, which act as central sentinels and amplifiers of systemic inflammation. In these cells, MyD88 engages IRF-5 to drive proinflammatory cytokine transcription (Takaoka et al., 2005), and its palmitoylation at C113 by ZDHHC6, dependent on fatty acid synthesis, couples lipid metabolism to innate activation (Kim et al., 2019). Along the gut-lung axis, intestinal ischemia-reperfusion triggers bacterial translocation, and MyD88 deficiency ablates both local gut injury and remote lung inflammation (Victoni et al., 2010). In sepsis, progressive myocardial upregulation of TLR2/3/4/7 and MyD88 (Mylonas et al., 2025), and in the kidney, tubular epithelial cell-specific deletion of MyD88 attenuates senescence and injury (Zhang et al., 2026b). Therapeutically, valproic acid-coupled nanoparticles suppress the TLR-MyD88-NFκB axis in primary human macrophages, outperforming free drug (Kuhne et al., 2021). Conversely, the gut microbe-derived metabolite hippuric acid selectively enhances MyD88-dependent pro-inflammatory responses in macrophages via cholesterol biosynthesis, and its elevated levels correlate with sepsis mortality (Mirji et al., 2026). Thus, macrophages serve as a central cellular hub linking gut microbial metabolites, lipid remodeling, and TLR-MyD88 signaling to distant organ injury, offering actionable therapeutic nodes.

### 4.4 AhR

Gut microbiota-derived tryptophan metabolites shape sepsis outcomes by activating the AhR in macrophages, establishing AhR as a central hub in microbiota-driven gut-organ crosstalk. In a *Serratia marcescens* sepsis model, microbiota-derived indoles activate macrophage AhR to enhance bacterial clearance and survival, whereas the pathogen-secreted siderophore enterobactin inhibits AhR and increases mortality (Keskey et al., 2025). Along the gut-lung axis, oral manganese-carbon dots enrich *Clostridium* and *Bacteroides*, elevating IPA; IPA binds AhR to promote efferocytosis and anti-inflammatory polarization of pulmonary macrophages, alleviating septic lung injury-an effect lost upon gut microbiota depletion (Peng et al., 2025). In the gut itself, ozonated water increases DL-tryptophan, activates AhR, and suppresses necroptosis in intestinal epithelial cells, protecting against sepsis-induced intestinal damage in a microbiota-dependent manner (Wang et al., 2025). CHAC1 deficiency remodels the gut microbiota, enriching *Akkermansia muciniphila* and indole-3-carboxylic acid (ICA); ICA activates macrophage AhR, shifting metabolism from glycolysis to oxidative phosphorylation, thereby conferring resistance to multi-organ injury (Hu et al., 2026). IPA also directly enhances macrophage phagocytosis via AhR, and lower fecal IPA levels in septic patients correlate with bacteremia and 28-day mortality, adding predictive value to the SOFA score (Huang et al., 2022). Collectively, these findings position AhR as a key checkpoint in macrophage-mediated gut-organ communication, offering a unifying therapeutic target for sepsis.

## 5 Host-directed therapy

The central role of macrophage immunometabolism in sepsis pathophysiology has stimulated growing interest in host-directed therapies, which modulate host immune responses rather than directly targeting pathogens. These approaches offer distinct advantages: effectiveness against diverse, including drug-resistant, pathogens; the potential to restore immune homeostasis without excessive immunosuppression; and compatibility with conventional antimicrobial regimens. 

### 5.1 Metabolic modulators

#### 5.1.1 Glycolysis inhibitors

Inhibition of glycolysis, a hallmark of M1-driven hyperinflammation, has been investigated in sepsis models. In a sepsis reinfection model, 2-DG ameliorated pulmonary inflammation by restraining TRIM in granulocytes through metabolic reprogramming. However, its nonspecific suppression of biofunctions necessitates caution (Li et al., 2025). Separately, 2-DG attenuated IL-6, TNF-α, and IL-1β signaling by preventing gp130 glycosylation, and demonstrated efficacy in colitis, arthritis, and LPS-induced shock models (Uehara et al., 2022). Furthermore, PKM2 has been identified as a convergent metabolic node that couples CARM1-driven glycolytic reprogramming to ferroptosis, suppresses NLRP3 inflammasome activation through platycodin D3 binding, and is stabilized by Scap to sustain antifungal macrophage immunity (Huang et al., 2026; Wang et al., 2026; Wang et al., 2026).

#### 5.1.2 Mitochondrial-targeted therapies

Preserving mitochondrial function represents a promising therapeutic strategy. Metformin, which inhibits complex I and reduces mtROS, suppresses the mtROS/cGAS-STING-IFN-β axis in macrophages, promotes mitophagy, and improves outcomes in preclinical models (Ji et al., 2025). Mitochondria-targeted antioxidants (e.g., MitoQ) attenuate oxidative damage and inflammation, while mitophagy enhancers such as urolithin A and rapamycin improve mitochondrial clearance and function in sepsis (Manfredini et al., 2019; Li et al., 2024; Mohsin et al., 2025; Sun et al., 2025).

#### 5.1.3 Sirtuin activators

Compounds that activate sirtuins, such as resveratrol and synthetic SIRT1 agonists, exert beneficial effects in preclinical models of sepsis (Li et al., 2018; You et al., 2024). SIRT1 activation suppresses NFκB signaling, whereas its inhibition reverses the macrophage M2-polarizing effects of nicotinamide mononucleotide (NMN) (Li et al., 2018; He et al., 2024). NAD^+^ precursors, including nicotinamide riboside and nicotinamide mononucleotide, replenish NAD^+^ levels to further sustain sirtuin activity, thereby improving mitochondrial function and survival in experimental sepsis (Hong et al., 2018; Cros et al., 2022; Li et al., 2023; Zhao et al., 2023).

### 5.2 Microbiota-based interventions

Restoring a healthy gut microbiota represents an attractive strategy to modulate macrophage function through metabolite-mediated pathways.

#### 5.2.1 Probiotics and prebiotics

Probiotic preparations containing beneficial bacteria (*Lactobacillus, Bifidobacterium* species) have been investigated in sepsis. By restoring SCFA production and competing with pathogens, probiotics support gut barrier and immunity, but responses vary and translocation risks limit use in immunocompromised patients (Lou et al., 2024; Ma et al., 2026). Prebiotics-dietary fibers that selectively promote beneficial bacteria-enhance SCFA production and safely restore immunomodulation (Castell et al., 2022; Arioz Tunc et al., 2026).

#### 5.2.2 Fecal microbiota transplantation

Fecal microbiota transplantation restores a healthy microbial ecosystem via donor stool. In preclinical sepsis models, it attenuates lung injury and improves survival (Tang et al., 2024; Zhao and Wu, 2025; Wang et al., 2026). Case reports in humans suggest potential benefit, but controlled trials are lacking (Qin et al., 2024; Yang et al., 2024). Safety concerns, particularly transmission of undetected pathogens, require careful consideration (Elendu et al., 2025; Berry et al., 2026).

#### 5.2.3 SCFA supplementation

Direct SCFA supplementation bypasses microbial production. Butyrate, propionate, and acetate have each shown protective effects in sepsis models, reducing inflammation, preserving barrier function, and improving outcomes (Yang et al., 2025a). Clinical development of SCFA-based therapies faces challenges related to formulation, pharmacokinetics, and target tissue delivery (Green et al., 2024; Zewail et al., 2025).

### 5.3 Cell-based and extracellular vesicle therapies

Bone marrow-derived mesenchymal stem cells (MSCs) have been widely investigated in preclinical sepsis models. They exert immunomodulatory effects primarily via paracrine mechanisms, releasing extracellular vesicles, growth factors, and anti-inflammatory mediators (Zheng et al., 2018; Premer et al., 2025). Latest research confirms that human placental mesenchymal stem cells (PMSCs) ameliorated severe pneumonia-induced ARDS by selectively preserving resident M2 alveolar macrophages (AMΦs) and enhancing their antibacterial functions while diminishing recruited M1 bone marrow-derived macrophages (Wang et al., 2023). Although clinical trials of MSCs in sepsis and ARDS have established a favorable safety profile, therapeutic efficacy has been inconsistent, likely due to variations in patient characteristics and cell-product preparation (Matthay et al., 2025). Refinements in donor selection, cell processing, dosing regimens, and patient stratification may enhance outcomes.

Extracellular vesicles (EVs) serve a dual role in sepsis, functioning as both pathogenic mediators and therapeutic candidates (Quaglia et al., 2022; You et al., 2025). Acting as natural shuttles, these vesicles carry bioactive molecules that can either promote inflammatory responses or serve as drug delivery vehicles (Cao et al., 2023; You et al., 2025). In their pro-inflammatory capacity, LPS-stimulated EVs carry miR-223-3p, which primes alveolar macrophages toward autophagy and ferroptosis through the MEF2C/Hippo axis (Ye et al., 2025). This process ultimately aggravates sepsis-related ALI. EVs propagate inflammatory signals and drive immunosuppression in sepsis (Schiavello et al., 2026). Conversely, MSC-derived EVs show therapeutic promise. MSC small EVs deliver miR-125a-5p to tumor necrosis factor receptor 2 (TNFR2), blocking tumor necrosis factor (TNF)-driven pyroptosis through NFκB/NLRP3 and alleviating septic acute kidney injury (Chen et al., 2026). Similarly, iMSC-EVs exhibit immunomodulatory and cytoprotective properties in septic patient-derived PBMCs, highlighting their potential as a cell-free therapy for re-establishing immune homeostasis in sepsis (Meng et al., 2026). EVs also possess diagnostic and prognostic value, as their profiles correlate with disease severity and the extent of organ dysfunction (Li et al., 2023; Park et al., 2025). The EV-coagulolytic balance (EV-CLB) independently predicts 90-day mortality in septic shock (Lacroix et al., 2025). Collectively, these findings establish EVs as a versatile platform for sepsis diagnosis, prognosis, and precision intervention (Repici et al., 2025).

### 5.4 Challenges and opportunities in host-directed therapy

The major challenge for host-directed therapy in sepsis is profound patient heterogeneity. Sepsis is a heterogeneous syndrome; a therapy effective in one subgroup may harm another, necessitating a precision medicine approach (Plata-Menchaca et al., 2026). Future strategies should stratify patients based on dynamic immune-metabolic profiles rather than applying uniform immunosuppression. This requires biomarkers capable of capturing the evolving host response, such as metabolomics (e.g., succinate, itaconate), transcriptomic signatures, immunophenotyping, mitochondrial function assays, and IgG N-glycome analysis (Liu et al., 2023; Moore et al., 2025; Papareddy et al., 2025; Standke et al., 2025; Wang et al., 2025; Shen et al., 2026). Integrating these into a multi-omics platform could enable real-time monitoring and adaptive therapy.

Optimal timing is critical due to sepsis’s dynamic progression-an intervention effective in the hyper-inflammatory phase may be detrimental during immunosuppression (Ovali and Percin, 2025). Although the “sepsis immune clock” provides a framework, personalized timing requires continuous patient assessment (Liu and Liang, 2025; Papatheodosiou et al., 2026). Given the pathophysiological complexity, combination therapies targeting multiple pathways are promising. Rational strategies may include metabolic modulators with antimicrobials, mitochondrial-targeted agents with immune-stimulants, and short-chain fatty acids with probiotics to restore microbiota-immune function (Martin-Gallausiaux et al., 2021; Xu et al., 2025; Zhao et al., 2025).

## 6 Future directions and limitations

### 6.1 Future directions

Advancing host-directed therapies for sepsis will require deeper insights into the spatiotemporal regulation of metabolic signaling networks (Li et al., 2025). A systems-level understanding of how microbial metabolites interact to shape macrophage function is also needed (Zeng et al., 2025; Shapiro et al., 2026). Emerging technologies-including single-cell multi-omics, spatial transcriptomics, organ-on-chip models, and machine learning-based integration of multi-omics and clinical data-will help dissect sepsis heterogeneity and identify predictive signatures (Liu and Liang, 2025; Shen et al., 2026; Silva et al., 2026; Uhlig et al., 2026). Ultimately, the goal of precision immune-metabolic editing-tailoring therapies to dynamically restore immune homeostasis-relies on real-time biomarker monitoring, pathophysiology-based patient stratification, and adaptive intervention strategies.

### 6.2 Key challenges and limitations

Several limitations currently constrain progress. Organ-specific macrophage metabolic responses in sepsis remain incompletely understood, hindering the development of targeted interventions. Translating the “immune clock” concept into clinical practice is limited by the absence of validated, real-time biomarkers for immune-metabolic phenotyping. Preclinical models often fail to adequately recapitulate the complexity and heterogeneity of human sepsis, which may affect the translatability of findings. Moreover, most candidate host-directed therapies are still in early-phase trials, with their efficacy and safety yet to be established in diverse patient populations.

## 7 Conclusions

Macrophages occupy a central position in sepsis pathophysiology, where they integrate immune-metabolic reprogramming with inter-organ crosstalk via gut-organ axis. The dynamic polarization continuum of macrophages, ranging from glycolytic pro-inflammatory states to oxidative reparative phenotypes, delineates potential therapeutic windows. Mitochondrial dysfunction couples metabolic dysregulation to sustained inflammation and organ injury through mitochondrial ROS and cGAS-STING signaling. A coherent mechanistic sequence is constituted by infection-triggered gut dysbiosis, which produces gut-derived signals that reprogram macrophage metabolism and eventually lead to organ-specific outcomes. This framework provides a mechanistic basis for the gut microbiome’s modulation of the host response to sepsis. Nevertheless, clinical translation of host-directed strategies targeting macrophage immune-metabolism remains limited by the lack of dynamic biomarkers for patient stratification, and ultimately requires the integration of mechanistic insights, translational models, and clinical validation to enable precision immune-metabolic editing.

## References

[B1] Arioz Tunc H. Calder P. C. Cait A. Dodd G. F. Gasaly Retamal N. Y. I. Guillemet D. . (2026). Impact of non-digestible carbohydrates and prebiotics on immunity, infections, inflammation and vaccine responses: a systematic review of evidence in healthy humans and a discussion of mechanistic proposals. Crit. Rev. Food Sci. Nutr. 66, 1–74. doi: 10.1080/10408398.2025.2514700 40516031

[B2] Bailey J. D. DM Nicol T. McNeill E. Shaw A. Chuaiphichai S. Hale A. . (2019). Nitric oxide modulates metabolic remodeling in inflammatory macrophages through TCA cycle regulation and itaconate accumulation. Cell Rep. 28, 218–230, e217. doi: 10.1016/j.celrep.2019.06.018 31269442 PMC6616861

[B3] Bauer S. R. Mourany L. Gunsalus P. R. Milinovich A. Kane-Gill S. L. Wang X. . (2025). Sepsis and septic shock case identification from electronic health records: an open-source workflow and comparison of cohorts by criteria. Crit. Care 29, 523. doi: 10.1186/s13054-025-05749-1 41388426 PMC12699874

[B4] Berry P. Tariq R. Pardi D. S. Khanna S. (2026). Effectiveness and safety of fecal microbiota transplantation for recurrent Clostridioides difficile infection in immunocompromised patients. Clin. Gastroenterol. Hepatol. 24, 949–973. doi: 10.1016/j.cgh.2025.06.043 40759389

[B5] Cai S. Guo P. Wang H. Fu S. Ding K. Liang X. . (2026). High humidity reprograms the gut mycobiome to promote Meyerozyma caribbica-derived syringic acid and attenuate sepsis-induced acute kidney injury. mSystems 11(7), e0171625. doi: 10.1128/msystems.01716-25 PMC13386831 42294869

[B6] Cao M. Shi M. Zhou B. Jiang H. (2023). An overview of the mechanisms and potential roles of extracellular vesicles in septic shock. Front. Immunol. 14, 1324253. doi: 10.3389/fimmu.2023.1324253 PMC10853337 38343439

[B7] Cao Y. He X. Liu Z. Miao L. Zhu B. (2025). The potential of melatonin in sepsis-associated acute kidney injury: Mitochondrial protection and cGAS-STING signaling pathway. Heliyon 11, e41501. doi: 10.1016/j.heliyon.2024.e41501 39850412 PMC11755053

[B8] Castell M. Walton G. E. Perez-Cano F. J. (2022). Editorial: Beyond probiotics: Dietary microbial modulators of the immune system - effects and mechanisms. Front. Nutr. 9, 852086. doi: 10.3389/fnut.2022.852086 35308277 PMC8927886

[B9] Chen F. Tang T. T. Chen Z. Q. Yang Q. Zhang Y. Zhang Y. L. . (2026). Mesenchymal stem cell-derived small extracellular vesicles suppress pyroptosis by delivering miR-125a-5p to improve acute kidney injury in sepsis. Cell. Death Discov. 12. doi: 10.1038/s41420-026-03143-6 42156367 PMC13357607

[B10] Chen F. W. N. Liao J. Jin M. Qu F. Wang C. . (2024). Esculetin rebalances M1/M2 macrophage polarization to treat sepsis-induced acute lung injury through regulating metabolic reprogramming. J. Cell. Mol. Me 28, e70178. doi: 10.1111/jcmm.70178 39535339 PMC11558263

[B11] Chen J. W. LC Li S. Wu S. W. Cai C. Lu M. Q. (2024). Sepsis-associated liver injury: mechanisms and potential therapeutic target. World J. Gastroenterol. 30, 4518–4522. doi: 10.3748/wjg.v30.i42.4518 39563749 PMC11572628

[B12] Chen X. Zhu Y. Xia L. Su S. Fan S. Lu Y. . (2025). Glutamine limits NLRP3 inflammasome activation and pyroptosis in macrophages by sustaining the IRG1/itaconate axis. FEBS J. 292, 6016–6034. doi: 10.1111/febs.70119 40296302

[B13] GBD 2021 Antimicrobial Resistance Collaborators . (2024). Global burden of bacterial antimicrobial resistance 1990-2021: a systematic analysis with forecasts to 2050. Lancet 404(10459), 1199–1226. doi: 10.1016/S0140-6736(24)01867-1 PMC11718157 39299261

[B14] Cros C. Margier M. Cannelle H. Charmetant J. Hulo N. Laganier L. . (2022). Nicotinamide mononucleotide administration triggers macrophages reprogramming and alleviates inflammation during sepsis induced by experimental peritonitis. Front. Mol. Biosci. 9, 895028. doi: 10.3389/fmolb.2022.895028 35832733 PMC9271973

[B15] Ding J. Zhang S. Zhang X. (2025). ODC1 loss upon KLF6 upregulation promotes macrophage pyroptosis and acute kidney injury in sepsis. Hum. Cell 38, 163. doi: 10.1007/s13577-025-01290-z 40962975

[B16] Domínguez-Andrés J. NB Li Y. Scicluna B. P. Gresnigt M. S. Arts R. J. W. . (2019). The itaconate pathway is a central regulatory node linking innate immune tolerance and trained immunity. Cell Metab. 29, 211–220, e215. doi: 10.1016/j.cmet.2018.09.003 30293776

[B17] Du Q. HF Zhang S. Lan X. Pan Y. Wang J. (2026). Current understanding and advances regarding the adipose-immune-metabolic axis in disease tolerance during sepsis. Front. Immunol. 17, 1755423. doi: 10.3389/fimmu.2026.1755423 41853269 PMC12992056

[B18] Elendu C. Omeludike E. K. Aregbesola E. T. Mordi P. Blewusi G. S. Ogidan A. O. . (2025). Fecal microbiota transplantation as a therapeutic modality for recurrent Clostridioides difficile infection: reviewing efficacy, safety, mechanisms of action, and outcomes. Ann. Med. Surg. (Lond) 87, 5829–5850. doi: 10.1097/ms9.0000000000003649 40901203 PMC12401380

[B19] Fan X. Q. FS Dong B. Y. Zhang C. M. Zuo J. Zhang D. W. . (2025). Recent advances in the interaction between acute respiratory distress syndrome and gut microbiota: A narrative review. Infect. Genet. Evol. 134, 105810. doi: 10.1016/j.meegid.2025.105810 40789381

[B20] Fang Y. Dou A. Zhang Y. Zhang Y. Gao Y. Xie K. (2026). Research on sepsis and metabolic reprogramming from 1998 to 2025: a bibliometric and visualized analysis. Shock 66, 285–302. doi: 10.1097/SHK.0000000000002714 PMC13384386 40986928

[B21] Fu Y. G.T. Loughran P. A. Li Y. Billiar T. R. Liu Y. . (2025). Roles of TLR4 in macrophage immunity and macrophage-pulmonary vascular/lymphatic endothelial cell interactions in sepsis. Commun. Biol. 8, 469. doi: 10.1038/s42003-025-07921-3 40119011 PMC11928643

[B22] Galvan-Pena S. O'Neill L. A. (2014). Metabolic reprograming in macrophage polarization. Front. Immunol. 5, 420. doi: 10.3389/fimmu.2014.00420 PMC4151090 25228902

[B23] Gao H. Ding M. Liu Y. Wang Y. Zhao S. Chen J. . (2025). Reprogramming immunity with itaconate: metabolic mechanisms and therapeutic perspectives. Inflamm. Res. 74, 128. doi: 10.1007/s00011-025-02087-4 40956430

[B24] Gao Y. Tian X. Zhang X. Milebe Nkoua G. D. Chen F. Liu Y. . (2023). The roles of tissue-resident macrophages in sepsis-associated organ dysfunction. Heliyon 9, e21391. doi: 10.1016/j.heliyon.2023.e21391 38027963 PMC10643296

[B25] Gao H. Wang K. Suarez J. A. Jin Z. Rocha K. C. E. Zhang D. . (2023). Gut lumen-leaked microbial DNA causes myocardial inflammation and impairs cardiac contractility in ageing mouse heart. Front. Immunol. 14, 1216344. doi: 10.3389/fimmu.2023.1216344 37520546 PMC10373503

[B26] Gioia S. Carnevale R. Tavano D. Overi D. Ridola L. Nardelli S. . (2023). Association between gut-derived endotoxins and porto-sinusoidal vascular disorder with portal hypertension. Aliment. Pharmacol. Ther. 58, 1205–1216. doi: 10.1111/apt.17727 37728001

[B27] Gorecki G. P. BA Novac M. B. Costea D. G. Costea D. O. Costea A. C. . (2025). Rethinking post-sepsis syndrome: linking cellular dysfunction to the clinical picture. Crit. Care 29, 418. doi: 10.1186/s13054-025-05491-8 41039512 PMC12492880

[B28] Green C. G. Ong M. L. Y. Rowland S. N. Bongiovanni T. James L. J. Clifford T. . (2024). Investigating serum concentration profiles of orally ingested short-chain fatty acid supplements. Food Funct. 15, 11525–11536. doi: 10.1039/d4fo04028g 39498577

[B29] Gu B. Jiang Y. Huang Z. Li H. Yu W. Li T. . (2024). MRG15 aggravates sepsis-related liver injury by promoting PCSK9 synthesis and secretion. Int. Immunopharmacol. 140, 112898. doi: 10.1016/j.intimp.2024.112898 39128417

[B30] Han Z. Fu J. Gong A. Ren W. (2025). Bacterial indole-3-propionic acid inhibits macrophage IL-1beta production through targeting methionine metabolism. Sci. China Life Sci. 68, 1118–1131. doi: 10.1007/s11427-024-2789-1 39825207

[B31] He Y. CX Zhong J. Lin C. Situ J. Liu B. . (2025). Glucocorticoid reduces mortality in LPS-induced sepsis mouse model by inhibiting JAK1/STAT3-mediated inflammatory response and restoring tricarboxylic acid cycle. Life Sci. 375, 123744. doi: 10.1016/j.lfs.2025.123744 40408938

[B32] He S. Jiang X. Yang J. Wu Y. Shi J. Wu X. . (2024). Nicotinamide mononucleotide alleviates endotoxin-induced acute lung injury by modulating macrophage polarization via the SIRT1/NF-kappaB pathway. Pharm. Biol. 62, 22–32. doi: 10.1080/13880209.2023.2292256 PMC10732210 38100537

[B33] Hobai I. A. (2023). Cardiomyocyte reprogramming in animal models of septic shock. Shock 59, 200–213. doi: 10.1097/SHK.0000000000002024 36730767

[B34] Hofmaenner D. A. Kleyman A. Press A. Bauer M. Singer M. (2022). The many roles of cholesterol in sepsis: A review. Am. J. Respir. Crit. Care Med. 205, 388–396. doi: 10.1164/rccm.202105-1197tr 34715007 PMC8886946

[B35] Hong G. Zheng D. Zhang L. Ni R. Wang G. Fan G. C. . (2018). Administration of nicotinamide riboside prevents oxidative stress and organ injury in sepsis. Free Radic. Biol. Med. 123, 125–137. doi: 10.1016/j.freeradbiomed.2018.05.073 29803807 PMC6236680

[B36] Hu J. J. Feng S. Y. Wu L. He S. Y. Chen X. F. Li G. H. . (2026). Chac1 deficiency confers sepsis resistance by enriching gut microbiota-derived indole-3-carboxylic acid to drive macrophage metabolic shifts. J. Adv. Res. 26, S2090-1232(26)00368-1. doi: 10.1016/j.jare.2026.04.065 42049093

[B37] Hu Q. Ren H. Li G. Wang D. Zhou Q. Wu J. . (2019). STING-mediated intestinal barrier dysfunction contributes to lethal sepsis. EBioMedicine 41, 497–508. doi: 10.2139/ssrn.3310612 PMC6443583 30878597

[B38] Huang Z. B. Hu Z. Lu C. X. Luo S. D. Chen Y. Zhou Z. P. . (2022). Gut microbiota-derived indole 3-propionic acid partially activates aryl hydrocarbon receptor to promote macrophage phagocytosis and attenuate septic injury. Front. Cell. Infect. Microbiol. 12, 1015386. doi: 10.3389/fcimb.2022.1015386 36299625 PMC9589056

[B39] Huang P. Liu Y. Li Y. Xin Y. Nan C. Luo Y. . (2025). Metabolomics- and proteomics-based multi-omics integration reveals early metabolite alterations in sepsis-associated acute kidney injury. BMC Med. 23, 79. doi: 10.1186/s12916-025-03920-7 39934788 PMC11818193

[B40] Huang J. Wang Y. Li F. Zhang N. Tian G. Guo D. . (2026). Scap stabilizes PKM2 to promote glycolysis and enhance anti-fungal immunity in macrophages. Cell Rep. 45, 117106. doi: 10.1016/j.celrep.2026.117106 41831231

[B41] Huang Z. B. ZG Lu C. X. Gong C. Gao X. Lin Y. . (2025). Gut microbiota-derived 3-indoleacetic acid confers a protection against sepsis-associated encephalopathy through microglial aryl hydrocarbon receptors. Exp. Neurol. 384, 115055. doi: 10.1016/j.expneurol.2024.115055 39547500

[B42] Jain H. Kumar A. Almousa S. Mishra S. Langsten K. L. Kim S. . (2024). Characterisation of LPS+ bacterial extracellular vesicles along the gut-hepatic portal vein-liver axis. J. Extracell. Vesicles 13, e12474. doi: 10.1002/jev2.12474 39001704 PMC11245684

[B43] Ji T. Zhao T. Long S. Z. Wei C. Z. Cheng D. Y. Chen J. . (2025). Microvesicle-transferred mitochondria trigger cGAS-STING and reprogram metabolism of macrophages in sepsis. Microbiol. Spectr. 13, e0078125. doi: 10.1128/spectrum.00781-25 40905697 PMC12502667

[B44] Keskey R. C. Xiao J. Hyoju S. Lam A. Kim D. Sidebottom A. M. . (2025). Enterobactin inhibits microbiota-dependent activation of AhR to promote bacterial sepsis in mice. Nat. Microbiol. 10, 388–404. doi: 10.1038/s41564-024-01882-9 39779878 PMC11905502

[B45] Kim Y. C. Lee S. E. Kim S. K. Jang H. D. Hwang I. Jin S. . (2019). Toll-like receptor mediated inflammation requires FASN-dependent MYD88 palmitoylation. Nat. Chem. Biol. 15, 907–916. doi: 10.1038/s41589-019-0344-0 31427815

[B46] Kim H. G. Rincon J. C. Efron P. A. Maile R. (2025). DAMP-driven trained immunity: metabolic and epigenetic reprogramming in critical illness and chronic inflammation. Front. Immunol. 16, 1669054. doi: 10.3389/fimmu.2025.1669054 41368629 PMC12682684

[B47] Kuhne M. Kretzer C. Lindemann H. Godmann M. Heinze T. Werz O. . (2021). Biocompatible valproic acid-coupled nanoparticles attenuate lipopolysaccharide-induced inflammation. Int. J. Pharm. 601, 120567. doi: 10.1016/j.ijpharm.2021.120567 33812975

[B48] G Gürtler L. Schramm W. Seitz R. (2025). Viral sepsis - pathophysiology and disease manifestation. Infection 53, 775–784. doi: 10.1007/s15010-025-02486-z PMC12137479 39961996

[B49] Lacroix R. Judicone C. Souab K. H. Bonifay A. Loundou A. Bouriche T. . (2025). The procoagulant and fibrinolytic balance of extracellular vesicles predicts mortality in septic shock patients. J. Extracell. Vesicles 14, e70073. doi: 10.1002/jev2.70073 40560804 PMC12190547

[B50] Lahariya R. Anand G. Kumari B. (2025). Trained immunity in sepsis: exploring the molecular link to long-term cardiometabolic disorders. Immunol. Res. 73, 139. doi: 10.1007/s12026-025-09698-3 41032210

[B51] Li L. Chen Z. Fu W. Cai S. Zeng Z. (2018). Emerging evidence concerning the role of sirtuins in sepsis. Crit. Care Res. Pract. 2018, 5489571. doi: 10.1155/2018/5489571 30533222 PMC6250024

[B52] Li N. GY Zhu Y. Li B. Wang C. Wang Z. . (2024). Exogenous acetate attenuates inflammatory responses through HIF-1α-dependent glycolysis regulation in macrophage. Cell. Mol. Life Sci. 82, 21. doi: 10.1007/s00018-024-05521-8 39725781 PMC11671453

[B53] Li F. Liu X. Yu Z. Zhang C. Tong S. Liu L. . (2025). 2-Deoxy-D-glucose reshapes the sepsis-trained stressed granulocytes by comprehensive alterations of the transcriptional landscape. Gene 972, 149828. doi: 10.1016/j.gene.2025.149828 41092992

[B54] Li H. R. Liu Q. Zhu C. L. Sun X. Y. Sun C. Y. Yu C. M. . (2023). Beta-nicotinamide mononucleotide activates NAD+/SIRT1 pathway and attenuates inflammatory and oxidative responses in the hippocampus regions of septic mice. Redox Biol. 63, 102745. doi: 10.1016/j.redox.2023.102745 37201414 PMC10206198

[B55] Li Q. Song X. C. Li K. Wang J. (2025). Gut-lung immunometabolic crosstalk in sepsis: From microbiota to respiratory failure. Front. Med. (Lausanne) 12, 1685044. doi: 10.3389/fmed.2025.1685044 41244772 PMC12613236

[B56] Li Y. TQ Liu C. Ma J. Chen Y. Wang L. Chen Y. . (2026). The metabolite α-ketoglutarate induces AIM2-dependent PANoptosis through demethylase TET2. Cell. Commun. Signal. 24, 193. doi: 10.1186/s12964-026-02740-3 41699684 PMC13014779

[B57] Li P. Wu Y. Goodwin A. J. Wolf B. Halushka P. V. Wang H. . (2023). Circulating extracellular vesicles are associated with the clinical outcomes of sepsis. Front. Immunol. 14, 1150564. doi: 10.3389/fimmu.2023.1150564 37180111 PMC10167034

[B58] Li J. Xuan M. Yang L. Liu Y. Lou N. Fu L. . (2025). Comprehensive single-cell analysis deciphered the immunoregulatory mechanism of TPPU in alleviating sepsis-related acute liver injury. J. Adv. Res. 71, 457–470. doi: 10.1016/j.jare.2025.02.018 39956402 PMC12126731

[B59] Li X. Zeng Q. Yao R. Zhang L. Kong Y. Shen B. (2024). Rapamycin mitigates organ damage by autophagy-mediated NLRP3 inflammasome inactivation in sepsis. Histol. Histopathol. 39, 1167–1177. doi: 10.14670/HH-18-706 38288570

[B60] Liang G. HJ Liu R. J. Chao Y. P. Hu Y. F. Zheng H. . (2024). α-Ketoglutarate plays an inflammatory inhibitory role by regulating scavenger receptor class a expression through N6-methyladenine methylation during sepsis. Eur. J. Immunol. 54, e2350655. doi: 10.1002/eji.202350655 38973083

[B61] Liao X. Wang Y. Zhang Z. Qu X. Zhou G. (2026). Bidirectional regulation of NLRP3 inflammasome and mitochondrial quality control in sepsis: mechanisms and therapeutic implications. Mediators Inflamm. 2026, 3168669. doi: 10.1155/mi/3168669 41613015 PMC12848910

[B62] Liu C. Cai Y. Ma Q. Sun W. (2026). Immunometabolic reprogramming in sepsis: mechanisms, clinical endotypes, and therapeutic opportunities. Front. Immunol. 17, 1731995. doi: 10.3389/fimmu.2026.1731995 41983123 PMC13070790

[B63] Liu D. Langston J. C. Prabhakarpandian B. Kiani M. F. Kilpatrick L. E. (2023). The critical role of neutrophil-endothelial cell interactions in sepsis: new synergistic approaches employing organ-on-chip, omics, immune cell phenotyping and in silico modeling to identify new therapeutics. Front. Cell. Infect. Microbiol. 13, 1274842. doi: 10.3389/fcimb.2023.1274842 PMC10800980 38259971

[B64] Liu H. Liang Q. (2025). Single-cell multi-omics-based immune temporal network resolution in sepsis: unravelling molecular mechanisms and precise therapeutic targets. Front. Immunol. 16, 1616794. doi: 10.3389/fimmu.2025.1616794 40861459 PMC12375439

[B65] Liu S. Y. Ruan H. Li S. S. (2025). HIF-1alpha: A bridge connecting sepsis and acute respiratory distress syndrome. Eur. J. Med. Res. 30, 827. doi: 10.1186/s40001-025-03107-z 40887582 PMC12399009

[B66] Liu T. Wen Z. Shao L. Cui Y. Tang X. Miao H. . (2023). ATF4 knockdown in macrophage impairs glycolysis and mediates immune tolerance by targeting HK2 and HIF-1alpha ubiquitination in sepsis. Clin. Immunol. 254, 109698. doi: 10.1016/j.clim.2023.109698 37481013

[B67] Liu Y. C. Yao Y. Yu M. M. Gao Y. L. Qi A. L. Jiang T. Y. . (2022). Frequency and mortality of sepsis and septic shock in China: a systematic review and meta-analysis. BMC Infect. Dis. 22, 564. doi: 10.1186/s12879-022-07543-8 35729526 PMC9210671

[B68] Lou J. Cui S. Huang N. Jin G. Chen C. Fan Y. . (2024). Efficacy of probiotics or synbiotics in critically ill patients: A systematic review and meta-analysis. Clin. Nutr. ESPEN 59, 48–62. doi: 10.1016/j.clnesp.2023.11.003 38220407

[B69] Luo X. Zhao Y. Luo Y. Lai J. Ji J. Huang J. . (2024). Cytosolic mtDNA-cGAS-STING axis contributes to sepsis-induced acute kidney injury via activating the NLRP3 inflammasome. Clin. Exp. Nephrol. 28, 375–390. doi: 10.1007/s10157-023-02448-5 38238499

[B70] Ma J. Jiang G. Hong L. Luo L. (2026). Global clinical trial landscape of microbiome modulator therapy in sepsis: gut microbiota interventions and challenges. Int. J. Surg. doi: 10.1097/js9.0000000000004472 41570295

[B71] Ma C. Liu S. Zhao Y. Liu H. Zhang X. (2025). DANCR promotes septic cardiomyopathy by enhancing macrophage glycolytic reprogramming via the IGF2BP2/HK2 axis. Front. Cell Dev. Biol. 13, 1628915. doi: 10.3389/fcell.2025.1628915 41394397 PMC12695775

[B72] Malla S. SN Saha (2025). Immunometabolism at the crossroads of infection: mechanistic and systems-level perspectives from host and pathogen. Immunometabolism (Cobham) 7, e00069. doi: 10.1097/in9.0000000000000069 41159060 PMC12558011

[B73] Manfredini A. C.L. Pinto M. C. Michels M. Burger H. Kist L. W. . (2019). Mitochondrial dysfunction is associated with long-term cognitive impairment in an animal sepsis model. Clin. Sci. (Lond) 133, 1993–2004. doi: 10.1042/cs20190351 31527095

[B74] Martin-Gallausiaux C. Marinelli L. Blottiere H. M. Larraufie P. Lapaque N. (2021). SCFA: mechanisms and functional importance in the gut. Proc. Nutr. Soc 80, 37–49. 10.1017/S0029665120006916 32238208

[B75] Maruyama D. Tian X. Doan T. N. M. Liao W. I. Chaki T. Taenaka H. . (2026). Gut microbiome-derived propionate reprograms alveolar macrophages metabolically and regulates lung injury responses in mice. Gut Microbes 18, 2606486. 10.1080/19490976.2025.2606486 PMC12758369 41467904

[B76] Matthay M. A. ZH Sarma A. Alipanah-Lechner N. Hendrickson C. Kornblith L. Z. Schreiber M. . (2025). Treatment with allogenic mesenchymal stromal cells for moderate to severe acute respiratory distress syndrome: a double-blind, placebo-controlled, multi-center, phase 2b clinical trial (STAT). Am. J. Respir. Crit. Care Med. 212(3), 428–439. doi: 10.1164/rccm.202411-2254oc 40728562 PMC12360293

[B77] Meng Y. Kong K. W. Chang Y. Q. Deng X. M. Yang T. (2023). Histone methyltransferase SETD2 inhibits M1 macrophage polarization and glycolysis by suppressing HIF-1alpha in sepsis-induced acute lung injury. Med. Microbiol. Immunol. 212, 369–379. doi: 10.1007/s00430-023-00778-5 37658121

[B78] Meng L. Liu Y. Wang N. Li T. Wang Y. Li M. (2026). Gut barrier-microbiota crosstalk in sepsis: from pathogenesis to potential therapies. Front. Immunol. 17, 1865498. doi: 10.3389/fimmu.2026.1865498 42396442 PMC13322907

[B79] Meng Q. Song Y. Wang C. Novak A. Taneja N. Ma Z. . (2026). Mesenchymal stem cell - derived extracellular vesicles modulate immune function in sepsis. Front. Immunol. 17, 1881925. doi: 10.3389/fimmu.2026.1881925 42523614 PMC13407162

[B80] Mirji G. Bhat S. A. El Sayed M. Reiser S. K. Gavara S. P. Ye Y. . (2026). Aromatic microbial metabolite hippuric acid enhances inflammatory responses in macrophages via TLR-MyD88 signaling and lipid remodeling. Cell Rep. 45, 116749. doi: 10.1016/j.celrep.2025.116749 41477766 PMC12884656

[B81] Misheva M. K.K. Davies L. C. Tyrrell V. J. Rodrigues P. R. S. Benavides G. A. . (2022). Oxylipin metabolism is controlled by mitochondrial β-oxidation during bacterial inflammation. Nat. Commun. 13, 139. doi: 10.1038/s41467-021-27766-8 35013270 PMC8748967

[B82] Miyamoto Y. Kikuta J. Matsui T. Hasegawa T. Fujii K. Okuzaki D. . (2024). Periportal macrophages protect against commensal-driven liver inflammation. Nature 629, 901–909. doi: 10.1038/s41586-024-07372-6 38658756

[B83] Mohsin M. Zaki A. Tabassum G. Khan S. Ali S. Ahmad T. . (2025). Urolithin-A supplementation alleviates sepsis-induced acute lung injury by reducing mitochondrial dysfunction and modulating macrophage polarization. Mitochondrion 84, 102047. doi: 10.1016/j.mito.2025.102047 40328344

[B84] Moore A. R. Zheng H. Ganesan A. Hasin-Brumshtein Y. Maddali M. V. Levitt J. E. . (2025). A consensus immune dysregulation framework for sepsis and critical illnesses. Nat. Med. 31, 4084–4096. doi: 10.1038/s41591-025-03956-5 41028543 PMC12705441

[B85] Murao A. Jha A. Kato T. Shimizu J. Akama Y. Aziz M. . (2025). Gut-primed neutrophils activate Kupffer cells to promote hepatic injury in mouse sepsis. Nat. Commun. 16, 9657. doi: 10.1038/s41467-025-65572-8 41213940 PMC12603279

[B86] Mylonas K. S. Peroulis M. Schizas D. Boletis J. N. Kapelouzou A. (2025). Upregulation of TLR-MYD88 signaling, stem cell genes, and Klotho mediates sepsis-associated myocardial inflammation. Mol. Biol. Rep. 52, 586. doi: 10.1007/s11033-025-10699-6 40504431

[B87] Ney L. M. Wipplinger M. Grossmann M. Engert N. Wegner V. D. Mosig A. S. (2023). Short chain fatty acids: key regulators of the local and systemic immune response in inflammatory diseases and infections. Open Biol. 13, 230014. doi: 10.1098/rsob.230014 36977462 PMC10049789

[B88] Novakovic B. Habibi E. Wang S. Y. Arts R. J. W. Davar R. Megchelenbrink W. . (2016). Beta-glucan reverses the epigenetic state of LPS-induced immunological tolerance. Cell. 167, 1354–1368, e1314. doi: 10.1016/j.cell.2016.09.034 27863248 PMC5927328

[B89] O'Neill L. A. Kishton R. J. Rathmell J. (2016). A guide to immunometabolism for immunologists. Nat. Rev. Immunol. 16, 553–565. 10.1038/nri.2016.70 PMC5001910 27396447

[B90] Ovali M. A. Percin S. (2025). Sepsis-associated immunosuppression: mechanistic insights, biomarkers, and therapeutic perspectives. Mol. Biol. Rep. 53, 148. doi: 10.1007/s11033-025-11312-6 41324739

[B91] Overi D. PN Carnevale R. Nardelli S. Tavano D. Ridola L. . (2025). Role of gut-derived endotoxins in porto-sinusoidal vascular disorder: comparison between patients with and without portal hypertension. Liver Int. 45, e70277. doi: 10.2139/ssrn.5177626 PMC12334859 40781803

[B92] Owen A. Patel J. M. Parekh D. Bangash M. N. (2022). Mechanisms of post-critical illness cardiovascular disease. Front. Cardiovasc. Med. 9, 854421. doi: 10.3389/fcvm.2022.854421 35911546 PMC9334745

[B93] Papareddy P. Lobo T. J. Holub M. Bouma H. Maca J. Strodthoff N. . (2025). Transforming sepsis management: AI-driven innovations in early detection and tailored therapies. Crit. Care 29, 366. doi: 10.1186/s13054-025-05588-0 40830514 PMC12366378

[B94] Papatheodosiou D. Giamarellos-Bourboulis E. J. Tsoukas C. (2026). Current status of immunomodulatory therapies in adult sepsis patients: where do we stand? Expert Rev. Anti Infect. Ther. 24, 239–257. doi: 10.1080/14787210.2026.2646192 41830448

[B95] Park C. Ryu T. Mohamed-Hinds R. Kim K. Kim J. H. Zou L. . (2025). Proteomic profiling of plasma extracellular vesicles identifies signatures of innate immunity, coagulation, and endothelial activation in septic patients. Sci. Rep. 15, 21871. doi: 10.1038/s41598-025-06430-x 40596376 PMC12215025

[B96] Payal N. Saxena J. Srivastava V. K. Kaushik S. Siddiqui A. J. Chauhan K. . (2025). Sepsis-associated brain dysfunction: Mechanisms, clinical insights, and therapeutic strategies. Curr. Drug Targets 26, 992–1009. doi: 10.2174/0113894501381183250825144834 40908529

[B97] Peng L. Song H. Shi H. Wu L. Ma Y. Fan X. . (2025). Oral multi-enzymatic manganese-carbon dots alleviate sepsis-associated lung injury via the gut-lung axis. ACS Nano 19, 37758–37782. doi: 10.1021/acsnano.5c10625 41128412 PMC12593376

[B98] Pereira-Dutra F. S. S.J. Souza E. K. Savi R. V. Souza T. S. Gil H. . (2026). Sepsis-induced lipid droplet accumulation enhances antibacterial innate immunity through mechanisms dependent on DGAT-1 and interferon-beta. Metabolism 174, 156389. doi: 10.1016/j.metabol.2025.156389 40972976

[B99] Pires S. Longman R. S. (2026). Emergency granulopoiesis and innate immune memory in inflammatory bowel disease. Trends Immunol. 5, S1471-4906(26)00183-3. doi: 10.1016/j.it.2026.07.004 42557141 PMC13449242

[B100] Plata-Menchaca E. P. Iba T. Ferrer R. (2026). Update on sepsis and septic shock: from bundles to precision medicine. Intensive Care Med. 52, 324–327. doi: 10.1007/s00134-025-08291-4 41586886

[B101] Premer C. HJ Yuan S. Y. Wilson J. W. (2025). Mesenchymal stem/stromal cells as a therapeutic for sepsis: a review on where do we stand? Stem Cell Res. Ther. 16, 245. doi: 10.1186/s13287-025-04371-w 40375314 PMC12082945

[B102] Qian S. Su Z. Lin J. Hou Q. Wang X. Li Y. . (2025). Inhibition of Farnesoid-x-receptor signaling during abdominal sepsis by dysbiosis exacerbates gut barrier dysfunction. Cell. Commun. Signal. 23, 236. doi: 10.1186/s12964-025-02224-w 40399878 PMC12096737

[B103] Qin H. Fu Y. Deng C. Chen Y. Huang K. Ruan Y. . (2024). The role of gut microbiota and the gut-lung axis in sepsis: A case study of a pregnant woman with severe rickettsial pneumonia and septic shock complicated by MODS. Clin. Case Rep. 12, e8815. doi: 10.1002/ccr3.8815 38840756 PMC11150134

[B104] Quaglia M. Fanelli V. Merlotti G. Costamagna A. Deregibus M. C. Marengo M. . (2022). Dual role of extracellular vesicles in sepsis-associated kidney and lung injury. Biomedicines 10. doi: 10.3390/biomedicines10102448 36289710 PMC9598620

[B105] Rao M. McGonagill P. W. Brackenridge S. Remy K. E. Caldwell C. C. Hotchkiss R. S. . (2025). Functional immunophenotyping for precision therapies in sepsis. Shock 63, 189–201. doi: 10.1097/SHK.0000000000002511 PMC12447363 39617419

[B106] Repici A. PG Wolfe V. Kaplan J. Nakamura T. Zingarelli B. (2025). Role of extracellular vesicles as mediators of cell communication and novel biomarkers in sepsis. J. Clin. Med. 14, 6649. doi: 10.3390/jcm14186649 41010852 PMC12470909

[B107] Ruan D. Yan X. Tang Y. Yang S. Yang X. Zhang M. . (2026). The endogenous metabolite TDCA ameliorates LPS-driven liver injury via modulation of caspase-11/GSDMD-mediated pyroptosis. Int. J. Mol. Sci. 27. doi: 10.3390/ijms27052273 41828497 PMC12985336

[B108] Rudd K. E. Johnson S. C. Agesa K. M. Shackelford K. A. Tsoi D. Kievlan D. R. . (2020). Global, regional, and national sepsis incidence and mortality, 1990-2017: analysis for the Global Burden of Disease Study. Lancet 395, 200–211. doi: 10.1016/s0140-6736(19)32989-7 31954465 PMC6970225

[B109] Saavedra-Torres J. S. Pinzon-Fernandez M. V. Nati-Castillo H. A. Cadena Correa V. Lopez Molina L. C. Gaitan J. E. . (2025a). Immunodynamic disruption in sepsis: mechanisms and strategies for personalized immunomodulation. Biomedicines 13. doi: 10.3390/biomedicines13092139 41007702 PMC12467355

[B110] Saavedra-Torres J. S. Pinzon-Fernandez M. V. Ocampo-Posada M. Nati-Castillo H. A. Jimenez Hincapie L. A. Cadrazo-Gil E. J. . (2025b). Inflammasomes and signaling pathways: key mechanisms in the pathophysiology of sepsis. Cells 14. doi: 10.3390/cells14120930 40558557 PMC12191029

[B111] Santana K. G. Righetti R. F. Breda C. N. S. Dominguez-Amorocho O. A. Ramalho T. Dantas F. E. B. . (2021). Cholesterol-ester transfer protein alters M1 and M2 macrophage polarization and worsens experimental elastase-induced pulmonary emphysema. Front. Immunol. 12, 684076. doi: 10.3389/fimmu.2021.684076 34367144 PMC8334866

[B112] Sarkar A. Mitra P. Lahiri A. Das T. Sarkar J. Paul S. . (2023). Butyrate limits inflammatory macrophage niche in NASH. Cell Death Dis. 14, 332. doi: 10.1038/s41419-023-05853-6 37202387 PMC10195803

[B113] Schiavello M. VB Bosco O. Dini C. Pivetta E. Morello F. Lupia E. . (2026). Decoding immune dysregulation in sepsis through extracellular vesicles: a path to precision medicine. Pharmaceutics 18, 570. doi: 10.3390/pharmaceutics18050570 42198264 PMC13210161

[B114] Shapiro H. Jickeli B. Niv I. Ernan B. Elinav E. (2026). The interplay between the microbiome and immune cells in metabolic homeostasis and disease. Cell Metab. 38(10), 1985–2005. doi: 10.1016/j.cmet.2026.06.004 42372727

[B115] Shen Y. Zhang P. Luo J. Chen S. Gu S. Lin Z. . (2026). Artificial intelligence drives advances in multi-omics analysis and precision medicine for sepsis. Biomedicines 14(2), 261. doi: 10.3390/biomedicines14020261 41751160 PMC12937605

[B116] Shimizu J. Murao A. Nofi C. Wang P. Aziz M. (2022). Extracellular CIRP promotes GPX4-mediated ferroptosis in sepsis. Front. Immunol. 13, 903859. doi: 10.3389/fimmu.2022.903859 35844517 PMC9277504

[B117] Silva M. J. Fehrenbach G. W. Pogue R. Murray P. Rezoagli E. Laffey J. G. . (2026). Sepsis modelling: current approaches and organ-on-chip perspectives. EBioMedicine 125, 106120. doi: 10.1016/j.ebiom.2026.106120 41690250 PMC12925287

[B118] Song H. Ma Y. Peng L. Gao F. Fan X. Yang M. . (2025). Platinum-doped emodin carbon dots mitigate sepsis-induced lung injury by targeting the gut-lung axis. J. Nanobiotechnology 24, 84. doi: 10.1186/s12951-025-03972-0 41454342 PMC12849688

[B119] Souza-Moreira L. Soares V. C. Dias S. Bozza P. T. (2019). Adipose-derived mesenchymal stromal cells modulate lipid metabolism and lipid droplet biogenesis via AKT/mTOR -PPARgamma signalling in macrophages. Sci. Rep. 9, 20304. doi: 10.1038/s41598-019-56835-8 31889120 PMC6937267

[B120] Standke A. Neu C. Baumbach P. Plooij A. K. Skitek K. Gotze J. . (2025). Reversible impairment of non-invasively assessed mitochondrial oxygen metabolism in the long-term course of patients with sepsis: a prospective monocentric cohort study. Intensive Care Med. Exp. 13, 114. doi: 10.1186/s40635-025-00808-x 41233688 PMC12615860

[B121] Strich J. R. Teague H. L. Yao X. Cruz R. S. Childs R. W. (2026). Breaking the stalemate: advancing sepsis therapeutics beyond supportive care. Public Health Rep., 333549251403358. doi: 10.1177/00333549251403358 41527845 PMC12799469

[B122] Sun J. Jin S. Wang Z. (2025). MitoQ alleviates mitochondria damage in sepsis-acute lung injury in a citrate synthase dependent manner. Inflamm. Res. 74, 92. doi: 10.1007/s00011-025-02055-y 40553170 PMC12187896

[B123] Sun X. Liu Y. Wang J. Zhang M. Wang M. (2022). Cardioprotection of M2 macrophages-derived exosomal microRNA-24-3p/Tnfsf10 axis against myocardial injury after sepsis. Mol. Immunol. 141, 309–317. doi: 10.1016/j.molimm.2021.11.003 34933177

[B124] Sun J. Zhang J. Wang X. Ji F. Ronco C. Tian J. . (2020). Gut-liver crosstalk in sepsis-induced liver injury. Crit. Care 24, 614. doi: 10.1186/s13054-020-03327-1 33076940 PMC7574296

[B125] Sviridov D. Netea M. G. Bukrinsky M. I. (2025). Maladaptive trained immunity in viral infections. J. Clin. Invest. 135. doi: 10.1172/jci192469 40892509 PMC12404746

[B126] Takaoka A. Yanai H. Kondo S. Duncan G. Negishi H. Mizutani T. . (2005). Integral role of IRF-5 in the gene induction programme activated by Toll-like receptors. Nature 434, 243–249. doi: 10.1038/nature03308 15665823

[B127] Tan C. GJ Chen H. Li T. Deng H. Liu K. . (2020). Inhibition of aerobic glycolysis promotes neutrophil to influx to the infectious site via CXCR2 in sepsis. Shock 53, 114–123. doi: 10.1097/shk.0000000000001334 30829852

[B128] Tang Y. CL Yang J. Zhang S. Jin J. Wei Y. (2024). Gut microbes improve prognosis of Klebsiella pneumoniae pulmonary infection through the lung-gut axis. Front. Cell. Infect. Microbiol. 14, 1392376. doi: 10.3389/fcimb.2024.1392376 38903943 PMC11188585

[B129] Tang J. Sung H. K. Lei Y. Teerawongsuwan S. Rungsiwiwut R. Wannaiampikul S. . (2026a). ALY688 confers cardioprotection through macrophage TGFbeta-mediated Treg/Th17 dependent multi-organ responses. Pharmacol. Res. 231, 108359. doi: 10.1016/j.phrs.2026.108359 42521105

[B130] Tang J. Yan M. Liu Y. Li W. Hu Z. (2026b). Mechanisms and therapeutics of immunometabolic reprogramming driving macrophage-ECs interactions in sepsis-associated ARDS from the gut-lung axis perspective. Front. Immunol. 17, 1730799. doi: 10.3389/fimmu.2026.1730799 41846933 PMC12989369

[B131] Tang J. YM Li M. Hu Z. Zhou K. (2025). From "metabolic storm" to "immune paralysis": the dynamic evolution of macrophages and metabolism reprogramming in ARDS. Front. Immunol. 16, 1738713. 10.3389/fimmu.2025.1738713 PMC12758027 41488672

[B132] Tannahill G. M. Curtis A. M. Adamik J. Palsson-McDermott E. M. McGettrick A. F. Goel G. . (2013). Succinate is an inflammatory signal that induces IL-1β through HIF-1α. Nature 496, 238–242. doi: 10.1038/nature11986 PMC4031686 23535595

[B133] Tian Z. Li C. (2025). The role of gut microbiota in the onset and development of sepsis and its therapeutic potential: mechanisms and research progress. Front. Microbiol. 16, 1718549. doi: 10.3389/fmicb.2025.1718549 41472805 PMC12747673

[B134] Tian X. Yuan L. Zeng Y. (2025). Butyrate attenuates SA-AKI by inhibiting pyroptosis via the STING-GSDMD axis. Biochem. Biophys. Res. Commun. 743, 151143. doi: 10.1016/j.bbrc.2024.151143 39693943

[B135] Torres J. S. S. Tamayo-Giraldo F. J. Bejarano-Zuleta A. Nati-Castillo H. A. Quintero D. A. Ospina-Mejia M. J. . (2025). Sepsis and post-sepsis syndrome: a multisystem challenge requiring comprehensive care and management-a review. Front. Med. (Lausanne) 12, 1560737. doi: 10.3389/fmed.2025.1560737 40265185 PMC12011779

[B136] Tu Y. Liu J. Kong D. Guo X. Li J. Long Z. . (2023). Irisin drives macrophage anti-inflammatory differentiation via JAK2-STAT6-dependent activation of PPARgamma and Nrf2 signaling. Free Radic. Biol. Med. 201, 98–110. doi: 10.1016/j.freeradbiomed.2023.03.014 36940733

[B137] Uehara I. Kajita M. Tanimura A. Hida S. Onda M. Naito Z. . (2022). 2-Deoxy-d-glucose induces deglycosylation of proinflammatory cytokine receptors and strongly reduces immunological responses in mouse models of inflammation. Pharmacol. Res. Perspect. 10, e00940. doi: 10.1002/prp2.940 35212163 PMC8873284

[B138] Uhlig M. Schumacher D. Marx G. Seikrit C. Schmidt-Ott K. M. Saritas T. . (2026). Insights from integrative spatial transcriptomics in sepsis-associated acute kidney injury. Nat. Rev. Nephrol. doi: 10.1038/s41581-026-01107-8 42521826

[B139] Van den Bossche J. Baardman J. de Winther M. P. (2015). Metabolic characterization of polarized M1 and M2 bone marrow-derived macrophages using real-time extracellular flux analysis. J. Vis. Exp. 105, 53424. doi: 10.3791/53424 PMC4692751 26649578

[B140] Victoni T. C.F. Soares A. L. de Freitas A. Secher T. Guabiraba R. Erard F. . (2010). Local and remote tissue injury upon intestinal ischemia and reperfusion depends on the TLR/MyD88 signaling pathway. Med. Microbiol. Immunol. 199, 35–42. doi: 10.1007/s00430-009-0134-5 19941004

[B141] Wang L. Kuang X. Li H. Zhang Y. Tang D. Niu Z. . (2026). GDF15 attenuates sepsis-associated liver injury by regulating macrophage polarization via the AMPK/PKM2/HIF-1a pathway. Eur. J. Pharmacol. 1013, 178505. doi: 10.1016/j.ejphar.2025.178505 41443362

[B142] Wang H. LH Guo Z. Hou H. Hou H. Chen B. (2025). Immunoglobulin G N-Glycome as a biomarker of mortality risk in Escherichia coli induced sepsis. Front. Immunol. 16, 1532145. doi: 10.3389/fimmu.2025.1532145 40165956 PMC11955649

[B143] Wang Y. Li N. Zhang X. Horng T. (2021). Mitochondrial metabolism regulates macrophage biology. J. Biol. Chem. 297, 100904. doi: 10.1016/j.jbc.2021.100904 34157289 PMC8294576

[B144] Wang Q. Liu C. Z. Li B. T. Yu X. Q. Zhang J. Y. Wang Z. T. . (2025). Ozone controls the metabolism of tryptophan protecting against sepsis-induced intestinal damage by activating aryl hydrocarbon receptor. World J. Gastroenterol. 31, 105411. doi: 10.3748/wjg.v31.i17.105411 40521273 PMC12159987

[B145] Wang Y. Qian J. Tan Y. Peng R. Wang D. (2026). CARM1 drives sepsis-induced lung injury by coupling PKM2-governed metabolic rewiring to macrophage ferroptosis. Int. Immunopharmacol. 182, 116835. doi: 10.1016/j.intimp.2026.116835 42119233

[B146] Wang J. D. J. Suan E. Li S. S. Shelat V. G. (2025). Sepsis and the diverse organ-gastrointestinal tract axis. World J. Crit. Care Med. 14, 105547. doi: 10.5492/wjccm.v14.i4.105547 41377546 PMC12687073

[B147] Wang B. Wang Y. Hu Z. Peng S. Li N. Dan Z. . (2025). Enhancing Sirt1-mediated deacetylation of p62 with a self-assembling nanopeptide and resveratrol hydrogel to mitigate sepsis-induced inflammation. Phytomedicine 145, 157047. doi: 10.1016/j.phymed.2025.157047 40627928

[B148] Wang M. T. Wang D. Qi Y. P. Chen Q. Qin X. M. Wang T. . (2026). Fecal microbiota transplantation alleviates sepsis-induced acute lung injury by improving mitochondrial function. Korean J. Physiol. Pharmacol. 30(5), 395–405. doi: 10.4196/kjpp.25.304 41991237 PMC13527573

[B149] Wang S. Xiong L. Ruan Z. Gong X. Luo Y. Wu C. . (2023). Indole-3-propionic acid alleviates sepsis-associated acute liver injury by activating pregnane X receptor. Mol. Med. 29, 65. doi: 10.1186/s10020-023-00658-x 37208586 PMC10197241

[B150] Wang L. T. Yen B. L. Wang H. H. Chao Y. Y. Lee W. Huang L. Y. . (2023). Placental mesenchymal stem cells boost M2 alveolar over M1 bone marrow macrophages via IL-1beta in Klebsiella-mediated acute respiratory distress syndrome. Thorax 78, 504–514. doi: 10.1136/thoraxjnl-2021-217928 PMC10176360 35450943

[B151] Wang J. Y.L. Cui K. Zhao J. Yang M. (2026). Platycodin D3 protects against sepsis-associated acute lung injury through direct inhibition of the PKM2-NLRP3 axis. Cell. Signal. 142, 112443. doi: 10.1016/j.cellsig.2026.112443 41748044

[B152] Wang B. Zhu L. Jia B. Zhao C. Zhang J. Li F. . (2023). Sepsis induces non-classic innate immune memory in granulocytes. Cell Rep. 42, 113044. doi: 10.1016/j.celrep.2023.113044 37643085

[B153] Wei N. Dai S. Li W. Zhou J. Chen Y. (2025). Gut microbiota and sepsis-associated encephalopathy: Pathogenesis and precision therapies. Front. Neurosci. 19, 1596467. doi: 10.3389/fnins.2025.1596467 40697274 PMC12279707

[B154] Wei H. Meng X. Fan H. Xia T. Wang H. Yang Y. . (2026). PRC1 impairs sepsis host defense by suppressing NF-kappaB/JAK-STAT signaling and macrophage glycolytic reprogramming. Int. Immunopharmacol. 182, 116816. doi: 10.1016/j.intimp.2026.116816 42114309

[B155] Wei M. Yi X. Lin Z. Cai J. Chen S. Zhou F. . (2026). Ketogenic diet alleviates septic lung injury via microbial gut-lung axis. Cell Metab. 38, 493–511, e412. doi: 10.1016/j.cmet.2026.01.005 41643678

[B156] Wiersinga W. J. van der Poll T. (2022). Immunopathophysiology of human sepsis. EBioMedicine 86, 104363. doi: 10.1016/j.ebiom.2022.104363 36470832 PMC9783164

[B157] Wu K. Ma Y. Wang Y. Wei R. Fang D. Li W. (2026). Macrophages in septic cardiomyopathy: Heterogeneity, function, and therapy. Shock 65, 905–915. doi: 10.1097/SHK.0000000000002683 40705380

[B158] Wu R. Yu Z. Guo P. Xiang X. Zeng Y. Fu S. . (2025). Desulfovibrio vulgaris exacerbates sepsis by inducing inflammation and oxidative stress in multiple organs. Front. Microbiol. 16, 1574998. doi: 10.3389/fmicb.2025.1574998 40371102 PMC12075292

[B159] Wu Y. Z.Y. Yu Y. Wang X. Zhen Z. Yuan Y. . (2025). Small intestinal γδ T17 cells promote SAE through STING/C1q-induced microglial synaptic pruning in male mice. Nat. Commun. 16, 6779. doi: 10.1038/s41467-025-62181-3 40702081 PMC12287374

[B160] Xiao K. Sun Y. Song J. Li L. Mao W. Jiang C. (2023). Gut microbiota involved in myocardial dysfunction induced by sepsis. Microb. Pathog. 175, 105984. doi: 10.1016/j.micpath.2023.105984 36638851

[B161] Xiao D. Wang X. Liang W. Yang Y. Du Y. Liu C. . (2025). Convergence of sepsis-associated encephalopathy pathogenesis onto microglia. J. Transl. Med. 23, 622. doi: 10.1186/s12967-025-06635-8 40462103 PMC12131422

[B162] Xie B. Wang M. Zhang X. Zhang Y. Qi H. Liu H. . (2024). Gut-derived memory gammadelta T17 cells exacerbate sepsis-induced acute lung injury in mice. Nat. Commun. 15, 6737. doi: 10.1038/s41467-024-51209-9 39112475 PMC11306781

[B163] Xu Y. Fan Y. Peng J. Zhang X. Luo Q. Shui K. . (2026). TREM1 deficiency attenuates LPS-induced sepsis-associated acute kidney injury by modulating macrophage polarization. Int. J. Surg. 112, 3003–3014. doi: 10.1097/JS9.0000000000003836 41147766

[B164] Xu L. Hao W. Yang Y. Wang Y. Li Y. Gong Y. . (2026). Metabolic-epigenetic rewiring of CCR5(hi) monocytes sustains long-term trained immunity against lethal sepsis. Sci. Adv. 12, eaee1634. doi: 10.1126/sciadv.aee1634 42627899 PMC13496178

[B165] Xu Y. Wang J. Yuan R. Qin Z. Long K. Gao P. (2025). Targeting the immuno-inflammatory-microbial network: a key strategy for sepsis treatment. Front. Immunol. 16, 1575516. doi: 10.3389/fimmu.2025.1575516 40297590 PMC12034552

[B166] Yan S. Xie Y. Xv J. Wang K. Wang J. Cao Y. . (2026). Shengjiang San attenuates sepsis-induced acute intestinal injury via Lactobacillus murinus derived IAAld-mediated macrophage polarization through NF-kappaB and TGF-beta signaling. J. Ethnopharmacol. 357, 120910. doi: 10.1016/j.jep.2025.120910 41265501

[B167] Yang Z. Kao X. Zhang L. Huang N. Wang Y. Chen J. . (2026). Dachaihu decoction alleviates septic liver injury by modulating the intestinal barrier dysfunction and suppressing the NF-kappaB/NLRP3/Caspase-1 signaling pathway. Chin. Med. 21. doi: 10.1186/s13020-026-01420-1 42298689 PMC13270745

[B168] Yang L. Shang W. Chen D. Qian H. Zhang S. Pan X. . (2025a). Identifying propionate metabolism-related genes as biomarkers of sepsis development and therapeutic targets. Sci. Rep. 15, 24531. doi: 10.1038/s41598-025-06463-2 40628770 PMC12238408

[B169] Yang Y. Wang L. Zhuang T. Xu T. Ji M. Wang Q. (2024). Washed microbiota transplantation stopped recurrent sepsis in a patient with myelofibrosis: a case report. Gut Pathog. 16, 78. doi: 10.1186/s13099-024-00660-6 39731142 PMC11673363

[B170] Yang B. Wu J. Hou X. Bai T. Liu S. (2025). Memory in misfire: the gut microbiome-trained immunity circuit in inflammatory bowel diseases. Int. J. Mol. Sci. 26. doi: 10.3390/ijms26199663 41096928 PMC12524489

[B171] Yang F. Yang W. Chen M. Ye W. Zhang Y. Wei H. . (2025). PPARgamma agonist alleviates sepsis-related liver injury by modulating M1/M2 macrophage polarization via the PPARgamma/IkappaBalpha/NF-kappaB pathway. Life Sci. 379, 123881. doi: 10.1016/j.lfs.2025.123881 40816499

[B172] Yang L. Zhang S. Cui L. Zhang J. Zhang S. Zhang L. . (2025b). Xuanfei baidu decoction alleviated sepsis-induced ALI by modulating gut microbial homeostasis and promoting inflammation resolution: bioinformatics and experimental study. ACS Omega 10, 13105–13121. doi: 10.1021/acsomega.4c10575 40224467 PMC11983172

[B173] Yang K. Zhao Q. Sun Y. Lin L. Han X. (2025). Mitochondrial immunometabolism in sepsis: orchestrating macrophage polarization and dysfunction. Eur. J. Med. Res. 31, 36. doi: 10.1186/s40001-025-03590-4 41331882 PMC12777342

[B174] Yao Q. Liu T. Wen J. Yang Q. Li Y. Yan H. . (2025). SpyTag-PEGylated probiotics delivering IL-1Ra modulate gut-lung crosstalk to mitigate septic lung injury. J. Control Release 386, 114163. doi: 10.1016/j.jconrel.2025.114163 40854504

[B175] Ye R. Wei Y. Li J. Zhong Y. Chen X. Li C. (2025). Plasma-derived extracellular vesicles prime alveolar macrophages for autophagy and ferroptosis in sepsis-induced acute lung injury. Mol. Med. 31, 40. doi: 10.1186/s10020-025-01111-x 39901167 PMC11792199

[B176] You J. Li Y. Chong W. (2024). The role and therapeutic potential of SIRTs in sepsis. Front. Immunol. 15, 1394925. doi: 10.3389/fimmu.2024.1394925 38690282 PMC11058839

[B177] You B. Yang Y. Wei J. Zhou C. Dong S. (2025). Pathogenic and therapeutic roles of extracellular vesicles in sepsis. Front. Immunol. 16, 1535427. doi: 10.3389/fimmu.2025.1535427 39967672 PMC11832720

[B178] Yu T. Liu Z. Liu Y. Gao M. Guo F. Liu Y. . (2026). Simiao Yongan decoction alleviates sepsis-induced liver injury by modulating macrophage polarization via gut-derived ursodeoxycholic acid. Phytomedicine 158, 158363. doi: 10.1016/j.phymed.2026.158363 42284645

[B179] Yuan H. Li B. Shen C. Xie L. Hou F. (2026). Microbiota-immune crosstalk in pneumonia and acute lung injury: Mechanisms, evidence, and therapeutic opportunities. Microorganisms 14. doi: 10.3390/microorganisms14081758 42655103 PMC13515912

[B180] Zeng L. Qian Y. Cui X. Zhao J. Ning Z. Cha J. . (2025). Immunomodulatory role of gut microbial metabolites: mechanistic insights and therapeutic frontiers. Front. Microbiol. 16, 1675065. doi: 10.3389/fmicb.2025.1675065 41040871 PMC12484209

[B181] Zewail M. Abbas H. Sayed N. E. Abd-El-Azim H. (2025). Intradermal delivery of teriflunomide loaded emulsomes using hollow microneedles for effective minimally invasive psoriasis management. Eur. J. Pharm. Biopharm. 210, 114692. doi: 10.1016/j.ejpb.2025.114692 40081673

[B182] Zhang J. Chen S. Hu X. Huang L. Loh P. Yuan X. . (2024a). The role of the peripheral system dysfunction in the pathogenesis of sepsis-associated encephalopathy. Front. Microbiol. 15, 1337994. doi: 10.3389/fmicb.2024.1337994 38298892 PMC10828041

[B183] Zhang J. Jiang J. Wang B. Wang Y. Qian Y. Suo J. . (2024b). SAP130 released by ferroptosis tubular epithelial cells promotes macrophage polarization via Mincle signaling in sepsis acute kidney injury. Int. Immunopharmacol. 129, 111564. doi: 10.1016/j.intimp.2024.111564 38320352

[B184] Zhang X. Liu H. Hashimoto K. Yuan S. Zhang J. (2022). The gut-liver axis in sepsis: interaction mechanisms and therapeutic potential. Crit. Care 26, 213. doi: 10.1186/s13054-022-04090-1 35831877 PMC9277879

[B185] Zhang Y. N.W. Li J. Bai Y. Bai Y. Jiang Z. . (2026a). 650 nm red-light therapy attenuates sepsis-induced acute lung injury via adiponectin-mediated immune-metabolic reprogramming. Front. Immunol. 17, 1710363. doi: 10.3389/fimmu.2026.1710363 41659861 PMC12872544

[B186] Zhang L. Tang M. Falsaperla R. Zhang K. (2025). The role of microglia in sepsis-associated encephalopathy: A narrative review. Transl. Pediatr. 14, 2363–2373. doi: 10.21037/tp-2025-496 41141658 PMC12552163

[B187] Zhang X. Wang M. Wang Y. Li L. Wang S. Xiang Y. (2026). Trained immunity in monocytes and macrophages in atherosclerotic cardiovascular disease. Cardiovasc. Res. 122, 1290–1306. doi: 10.1093/cvr/cvag109 42135201

[B188] Zhang Y. Wang Y. Zhao Y. Yao C. Dong Y. Sun K. . (2026b). Inhibition of myD88 in tubular epithelial cells alleviates the cellular senescence in sepsis-associated acute kidney injury. Inflammation. 49(1), 163. doi: 10.1007/s10753-026-02526-2 42154076 PMC13364797

[B189] Zhang B. Zhang H. Cheng L. Wang N. (2026). Macrophage metabolic reprogramming: a central hub linking multicellular crosstalk to organ vulnerability in sepsis. Tissue Cell. 104(Pt 1), 103831. doi: 10.1016/j.tice.2026.103831 42541906

[B190] Zhao L. Huang Y. Ye Z. Chen W. Zhang N. Wen Z. . (2026). Short-chain fatty acids attenuate sepsis-induced gut dysbiosis and hippocampal neuroinflammation via NLRP6 inflammasome activation in mice. Int. J. Surg. 112, 570–582. doi: 10.1097/JS9.0000000000003554 PMC12825554 41159411

[B191] Zhao X. MT Yu J. Hu Y. (2026). Gut microbiota as a therapeutic target in sepsis-induced multi-organ dysfunction: a review. Front. Cell. Infect. Microbiol. 16, 1823125. doi: 10.3389/fcimb.2026.1823125 42404770 PMC13328463

[B192] Zhao Y. QY Huang C. Li C. Zhang W. Wang X. . (2025). Neutrophils loaded NAD+ impede TLR4/NF-κB/NLRP3 pathway for sepsis treatmen. Mater. Today Bio 34, 102168. doi: 10.1016/j.mtbio.2025.102168 40809343 PMC12345315

[B193] Zhao Z. Wu B. (2025). Gut microbiota dysbiosis aggravates sepsis-induced lung injury by promoting neutrophil extracellular traps and suppressing host integrin defense. Front. Microbiol. 16, 1699748. 10.3389/fmicb.2025.1699748 PMC12827662 41586375

[B194] Zhao G. J. Yang X. Y. Zhang C. Dong W. Dong F. B. Zhang J. . (2023). Supplementation with nicotinamide riboside attenuates T cell exhaustion and improves survival in sepsis. Shock 60, 238–247. doi: 10.1097/shk.0000000000002153 37314209 PMC10476598

[B195] Zhao F. YZ Zhang L. Qi Y. Sun Y. Wang S. . (2025). Emerging advancements in metabolic properties of macrophages within disease microenvironment for immune therapy. J. Innate Immun. 17, 320–340. doi: 10.1159/000546476 40499523 PMC12252386

[B196] Zhao S. Zhang J. Wang W. Liang S. Chen M. Shao R. . (2026). Butyrate-induced trained immunity enhances bacterial killing ability of head kidney macrophages in turbot (Scophthalmus maximus L.). Fish Shellfish Immunol. 171, 111165. doi: 10.1016/j.fsi.2026.111165 41611137

[B197] Zheng G. Huang R. Qiu G. Ge M. Wang J. Shu Q. . (2018). Mesenchymal stromal cell-derived extracellular vesicles: regenerative and immunomodulatory effects and potential applications in sepsis. Cell Tissue Res. 374, 1–15. doi: 10.1007/s00441-018-2871-5 29955951

[B198] Zheng L. Jia T. Li Y. Zhang Z. Su H. Zhang R. (2026). The interplay between gastrointestinal dysfunction and gut microbiota dynamics in sepsis. Front. Cell. Infect. Microbiol. 16, 1761536. doi: 10.3389/fcimb.2026.1761536 42079748 PMC13128604

[B199] Zhou Z. K. YM Shou S. T. Chai Y. F. Liu Y. C. (2025). Interaction between gut-heart axis in sepsis-induced cardiomyopathy. Pharmacol. Res. 217, 107806. doi: 10.1016/j.phrs.2025.107806 40449812

[B200] Zhu Y. CX Lu Y. Xia L. Fan S. Huang Q. . (2022). Glutamine mitigates murine burn sepsis by supporting macrophage M2 polarization through repressing the SIRT5-mediated desuccinylation of pyruvate dehydrogenase. Burns Trauma 10, tkac041. doi: 10.1093/burnst/tkac041 36601059 PMC9801296

[B201] Zhu S. He J. (2026). Inflammation-driven immune reprogramming in sepsis: from cytokine storm to immunoparalysis. Front. Immunol. 17, 1887033. doi: 10.3389/fimmu.2026.1887033 42495640 PMC13391346

